# High energy density electrolytes for H_2_/Br_2_ redox flow batteries, their polybromide composition and influence on battery cycling limits†

**DOI:** 10.1039/d0ra10721b

**Published:** 2021-01-28

**Authors:** Michael Küttinger, Jakub K. Wlodarczyk, Daniela Daubner, Peter Fischer, Jens Tübke

**Affiliations:** Department of Applied Electrochemistry, Fraunhofer Institute for Chemical Technology ICT Joseph-von-Fraunhofer-Straße 7 76327 Pfinztal Germany michael.kuettinger@gmx.de; Institute of Computational Physics (ICP), Zurich University of Applied Sciences (ZHAW) Wildbachstrasse 21 8400 Winterthur Switzerland

## Abstract

Hydrogen–bromine redox flow batteries (H_2_/Br_2_-RFB) are a promising stationary energy storage solution, offering energy storage densities up to 200 W h L^−1^. In this study, high energy density electrolytes of concentrated hydrobromic acid of up to 7.7 M are investigated. Particular polybromide ion (Br_2*n*+1_^−^; *n* = 1–3) concentrations in the electrolyte at different states of charge, their effect on the electrolytic conductivity and cell operation limits are investigated for the first time. The concentrations of individual polybromides in the electrolytes are determined by Raman spectroscopy. Tribromide (Br_3_^−^) and pentabromide (Br_5_^−^) are predominantly present in equal concentrations over the entire concentration range. Besides Br_3_^−^ and Br_5_^−^, heptabromide (Br_7_^−^) exists in the electrolyte solution at higher bromine concentrations. It is shown that polybromide equilibria and their constants of Br_3_^−^ and Br_5_^−^ from literature are not applicable for highly concentrated solutions. The conductivity of the electrolytes depends primarily on the high proton concentration. The presence of higher polybromides leads to lower conductivities. The solubility of bromine increases disproportionately with increasing bromide concentration, since higher polybromides such as Br_7_^−^ or Br_5_^−^ are preferably formed with increasing bromide concentration. Cycling experiments on electrolyte in a single cell are performed and combined with limitations due to electrolyte conductivity and bromine solubility. Based on these results concentrations of the electrolyte are defined for potential operation in a H_2_/Br_2_-RFB in the range 1.0 M < *c*(HBr) < 7.7 M and *c*(Br_2_) < 3.35 M, leading to a theoretical energy density of 196 W h L^−1^.

## Abstract 2

Polybromides formation in aqueous bromine electrolytes and influence on H_2_/Br_2_ redox flow battery performance is investigated the first time.

## Graphical abstract

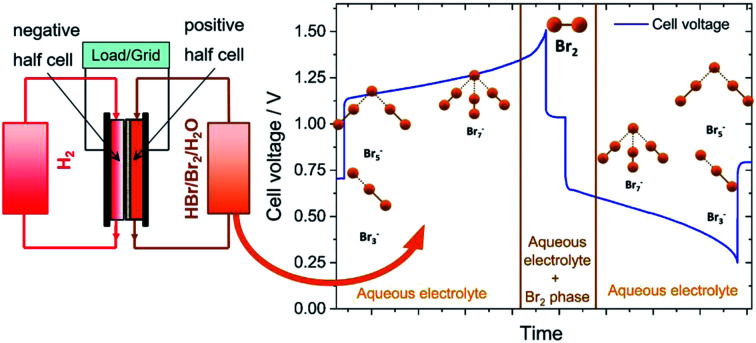

## Introduction

**Fig. 1:**
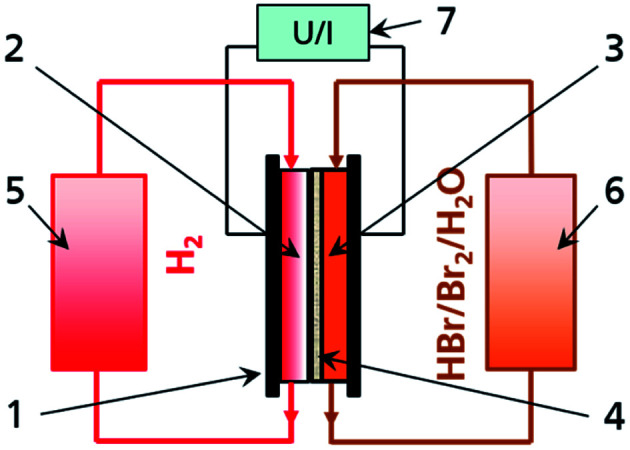
Principle of a H_2_/Br_2_ redox flow battery with (1) energy converter including (2) H_2_ negative half cell, (3) Br_2_/Br^−^ positive half cell and (4) membrane in the middle and media storage tanks for (5) H_2_ and (6) HBr/Br_2_/H_2_O electrolyte on left and right side and (7) grid connection.

The Br_2_/Br^−^ redox couple in aqueous solution is one of the most energy dense electrolytes in the positive half cell (Fig. 1) of redox flow batteries. The high energy density has been exploited in flow batteries like zinc–bromine RFB, sulphur–bromine RFB or hydrogen–bromine RFB (H_2_/Br_2_-RFB).^1–3^ The redox reaction in aqueous electrolyte in general converts two electrons per mole Br_2_ (eqn (1)):^4–6^

1 2Br^−^ ⇌ Br_2_ + 2e^−^

In this work, general composition and properties of high energy density electrolytes (theoretical 180 A h L^−1^/196 W h L^−1^) for H_2_/Br_2_-RFB are investigated in detail and the limits of their application in cell are provided by detailed analysis of polybromide equilibria and composition.

### State of the art

H_2_/Br_2_-RFB (Fig. 1) provide fast electrode kinetics in the negative hydrogen half cell and the positive bromine half cell and thus high power densities and energy efficiencies.^7^ High peak power densities of 1.4 W cm^−2^ and a voltage efficiency of 91% at 400 mW cm^−2^ were achieved in the past.^8^ Main research activities are the investigation of performant separators allowing for the limitation of hydrobromic acid (HBr), bromine (Br_2_) and polybromides crossover^8–12^ as well as the development of resistant catalyst materials.^13–15^ The H_2_/Br_2_-RFB storage technology is thereby progressing from prototype testing to commercialisation.^1,15^ For example, Enstorage demonstrated a 150 kW/900 kW h H_2_/Br_2_-RFB system^15^ and Elestor BV deployed field test systems.^16^ A recently published study predicts costs of $0.034 kW h^−1^ to $0.074 kW h^−1^ for large-scale 500 kW/5 MW h H_2_/Br_2_-RFB storage, and confirms competitiveness to fossil fuel energy generators and lithium based batteries.^16^ In H_2_/Br_2_-RFB studies, different electrolyte concentrations have been used in the past. Usually concentrations of bromine are below 2 M and concentration changes of HBr during operation are mainly below 4 M.^8,9,11,16,17^ Only capacities up to approximately 110 A h L^−1^ are utilised, although higher storage capacities are feasible with the electrolyte.

Aqueous HBr exhibits a high conductivity due to a high degree of dissociation.^18,19^ High concentrations of reactants lead to low mass transport limitation at high current densities. The maximum energy density of electrolytes is defined by the availability of high concentrated HBr. Commercially and industrially two types of HBr solutions of technical purity are available: HBr 48 wt% and HBr 62 wt% (Table 1). These high energy electrolytes exhibit theoretical volumetric capacities > 200 A h L^−1^ (Table 1). For the application in a battery, a dilution to 7.7 M HBr is more practical as it allows the mixture of HBr 48 wt% with further additives.

**Table 1:** Comparison of different commercial HBr electrolytes regarding their theoretical charge capacity and energy density

	Availability of technical electrolytes	
Mass concentration in wt%	HBr 62 wt%	HBr 48 wt%
Molar concentration *c*(HBr)/mol L^−1^	13.54^a^	8.84^a^
Theoretical charge capacity/A h L^−1^	362	237
Theoretical possible energy density^b^/W h L^−1^	395	258

a Calculated using product data of data sheets: density of solution, mass ratio of HBr and molar mass of HBr.

b Calculated with respect to the thermodynamic open circuit potential for the redox couple Br_2_/Br^−^ of 1.09 V (ref. 4, 19, 31 and 36) and based on Faraday law with a coulombic efficiency of 100%.

Bromine itself exhibits a limited solubility in pure water at around 0.214 mol L^−1^ at *θ* = 25 °C.^20–22^ The hydrolysis reaction of Br_2_ (ref. 23–26) is supressed in HBr for concentrations of 0.001 M or higher.^27–31^ Solubility of Br_2_ increases significantly in aqueous bromide solutions like hydrobromic acid. Soluble polybromide complexes (eqn (2) and (3)), such as tribromide (Br_3_^−^) or pentabromide ions (Br_5_^−^)^20,28,29^ are formed in an equilibrium reaction between Br_2_, bromide and the respective polybromide:

2 Br^−^ + Br_2_ ⇌ Br^−^_3_

3 Br^−^ + 2Br_2_ ⇌ Br^−^_5_

These are addition compounds between individual bromine molecules and the bromide ion.^32^ The formation of polybromides is advantageous, as high concentrations of HBr and Br_2_ can be combined. The equilibrium constants of the polybromide formation (*K*_*n*_) are determined according to the law of mass action, shown in eqn (4) and (5).^20,26–31,33–35^4
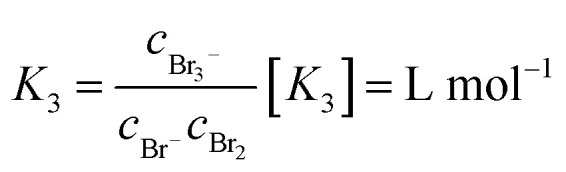
5
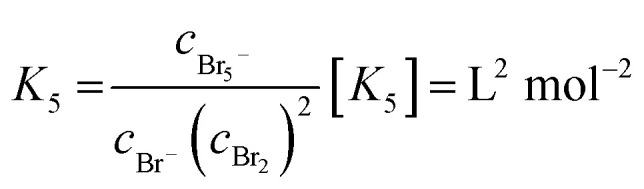


Values for *K*_3_ between 16.0 and 18.08 L mol^−1^ at 25 °C are known in literature.^20,26–31,33–35^ For *K*_5_, values between 19.45 L^2^ mol^−2^ and 40 L^2^ mol^−2^ are reported in literature.^20,29,31,33–35^ Both equilibria are shifted to the polybromide side and reduce free uncomplexed Br_2_ in the electrolyte.

In previous studies on polybromide equilibria, concentrations of bromide *c*(HBr) < 1.0 M were selected, while Br_2_ concentrations as low as *c*(Br_2_) < 240 mM were investigated.^20,26–31,33,34,37^ Furthermore, an experimental separation between Br_3_^−^ and Br_5_^−^ was only based on assumptions.^20^ An application of the known equilibria and their constants for the range of application of a bromine electrolyte up to *c*(HBr) = 7.7 M and *c*(Br_2_) = 3.35 M has not been investigated so far and seems to be doubtful, however, these are often adopted in studies on H_2_/Br_2_-RFB or Zn/Br_2_-RFB.^6,10,15,36,38–40^ Polybromides show Raman peaks in the range of the Raman shift^41–43^ between *ṽ* = 125 cm^−1^ and 325 cm^−1^. Raman peaks of symmetric and antisymmetric stretching vibrations of Br_3_^−^ and Br_5_^−^ as well as the symmetric stretching vibration of heptabromide (Br_7_^−^) at known Raman shifts^32,41–43^ can be considered and related to Br_2_ distribution and polybromide concentration. Although Raman data are accessible, no investigations on polybromide equilibria and electrolyte composition of highly concentrated acidic bromine electrolytes have been published so far. Neither the actual polybromide mixture nor its effect on relevant cell parameters are extensively investigated in the literature.

### Work plan

The limits of the applicability of 7.7 M HBr electrolytes in a H_2_/Br_2_ RFB single cell are shown in this work focusing on parameters like Br_2_ solubility and electrolyte conductivities. Operating conditions with the highly concentrated HBr/Br_2_ electrolytes are defined by introduction of a state of charge definition. Therefore, a new but comprehensive method for the determination of polybromide concentrations based on Raman spectroscopy for high concentrated polybromide electrolytes is presented. The existence of different polybromides in these samples is analysed, their concentrations are determined and their influence on the conductivity and application limits for highly concentrated HBr/Br_2_ electrolyte in a H_2_/Br_2_-RFB is investigated. Measured results on polybromide concentrations are compared with simulations based on literature values of the polybromide equilibrium constants of Br_3_^−^ and Br_5_^−^. The validity of the known equilibria is discussed.

## Experimental and methods

All experiments, setups, methods, suppliers and manufacturer data, and evaluation tools are presented in detail in the ESI.† Here, an overview of the methods is given.

### Chemicals and electrolyte preparation

Analysed electrolytes are composed of distilled water, hydrobromic acid HBr 48 wt% and bromine Br_2_. For this electrolyte investigation, an aqueous 7.7 M HBr solution is the initial electrolyte solution used, while also lower concentrations down to 0 M HBr are applied. This range is evaluated with 24 samples, with concentration changes in steps of Δ*c*(HBr) = −0.335 M and Δ*c*(Br _2_) = 0.167 M per sample. The correlation between Δ*c*(HBr) and Δ*c*(Br_2_) is stoichiometric and represented by eqn (1). The composition of all samples is given in Table 2. Each sample is used for the investigation of (i) electrolytic conductivity of the solution, (ii) detection of a possible miscibility gap of the solution and (iii) Raman investigations for polybromide detection as a basis for the calculation of polybromide concentrations. In addition, 24 samples with the same HBr concentrations, but without Br_2_, are mixed in order to investigate reference conductivities of pure HBr/H_2_O electrolytes. For a cell cycling test, a volume of 60 mL of *c*(HBr) = 7.7 M HBr in H_2_O is prepared.

Concentrations of HBr and Br_2_ in aqueous HBr/Br_2_/H_2_O electrolytes between 7.7 M HBr and 0 M HBr or 3.85 M Br_2_ and 0 M Br_2_ depending on the sample number. Concentrations show changes in electrolyte composition according to eqn (1). Table covers sample numbers 1 to 24

**Table:** 

Sample no.	1	2	3	4	5	6	7	8	9	10	11	12
*c*(HBr)/M	7.700	7.365	7.030	6.696	6.361	6.026	5.691	5.357	5.022	4.687	4.352	4.017
*c*(Br_2_)/M	0.000	0.167	0.335	0.502	0.670	0.837	1.004	1.172	1.339	1.507	1.674	1.841

**Table:** 

Sample no.	13	14	15	16	17	18	19	20	21	22	23	24
*c*(HBr)/M	3.683	3.348	3.013	2.678	2.343	2.009	1.674	1.339	1.004	0.670	0.335	0.000
*c*(Br_2_)/M	2.009	2.176	2.343	2.511	2.678	2.846	3.013	3.180	3.348	3.515	3.683	3.850

### Cell cycling test

**Fig. 2:**
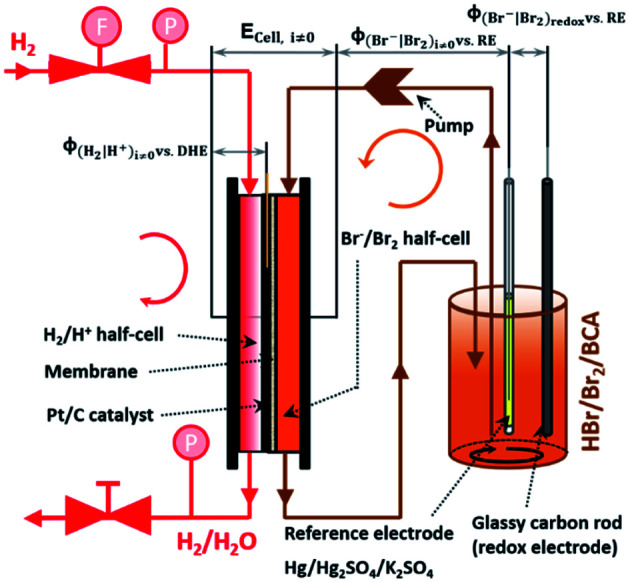
Experimental setup of the cell test of the investigated H_2_/Br_2_-RFB single cell including the possibility to measure voltages and potential differences: cell voltage during operation *E*_Cell *i*≠0_, potential of the positive half cell *φ*(Br_2_/Br^−^)_*i*≠0_*vs.* RE, potential of the negative half cell *φ*(H^+^/H_2_)_*i*≠0_*vs.* a dynamic hydrogen electrode DHE and the redox potential *φ*(Br_2_/Br^−^)_redox_ of the electrolyte at glassy carbon *vs.* RE.

A cell test is performed on a setup developed at Fraunhofer ICT. As membrane electrolyte assembly (MEA) a Nafion 117 membrane with single sided Pt/C catalyst for the hydrogen half cell is used. The gas diffusion electrode of the negative half cell consists of a gas diffusion layer (GDL) and a current collector with a milled meander structure. During the whole test the hydrogen half cell is fed with hydrogen externally and the hydrogen produced during the charging process is not stored. In the bromine half cell the electrode consists of a graphite felt, embedded in a frame, which is closed by a glassy carbon (GC) current collector. The cell is charged and discharged while a flow rate of 20 mL min^−1^ of electrolyte and different current densities of ±50 mA cm^−2^, ±100 mA cm^−2^, ±150 mA cm^−2^, ±200 mA cm^−2^ and ±250 mA cm^−2^ are applied. Voltage (*E*) thresholds of the experiments are between *E*_max_ = 1.5 V and *E*_min_ = 0.25 V. Cell voltage *E*_Cell *i*≠0_ under load, the redox potential *φ*(Br_2_/Br^−^)_redox_*versus* a normal hydrogen electrode (NHE) and half cell potentials of the positive bromine half cell *φ*(Br_2_/Br^−^)_*i*≠0_*vs.* NHE and of the negative hydrogen half cell *φ*(H^+^/H_2_)_*i*≠0_*vs.* NHE are determined in parallel during cycling experiments, as depicted in Fig. 2. A so-called “residual potential” Δ*E*_Res_ is calculated from these values as shown in eqn (6):

6 Δ*E*_Res_ = *E*_Cell *i*≠0_ − (*φ*(Br_2_/Br^−^)_*i*≠0_ − *φ*(H^+^/H_2_)_*i*≠0_)

It essentially reproduces the overvoltages between the negative and positive half cell, and represents the sum of electrolyte resistance and membrane resistance.

### Investigation of the miscibility

The prepared 24 HBr/Br_2_/H_2_O electrolyte samples are examined by visual inspection at *θ* = 23 °C for any phase separation of the aqueous phase and a further phase.

### Conductivity of electrolyte solutions

Electrolytic conductivities of electrolytes with HBr/Br_2_/H_2_O (24 samples) and electrolytes without Br_2_ in solution (24 samples), with their mixtures shown in Table 2 are examined at *θ* = 23 °C using a conductivity cell. Conductivities of the electrolytes are calculated from ohmic electrolyte resistances measured by helps of impedance spectroscopy. The cell constant of the conductivity cell is determined using 1 M KCl solution with a conductivity of 103.9 mS cm^−1^ at *θ* = 23 °C.^19^

### Solubility of Br_2_ in aqueous HBr solutions

A solubility curve of bromine in HBr solutions is determined by preparing aqueous HBr solutions of different concentrations *c*(HBr) between 0 M HBr and 5 M HBr at *θ* = 23 °C. Bromine is added dropwise with a pipette. The total mass of bromine is determined gravimetrically. This procedure is continued iteratively until a drop of bromine remains not dissolved in the sample. The addition of bromine increases the volume and mass of the sample solution. In order to determine the actually existing concentrations at saturation of *c*(HBr)_sat_ and *c*(Br_2_)_sat_, the volume and mass of the samples in the saturated state (*V*_sat._ and *m*_sat_., respectively) are measured, and concentrations at saturation are calculated.

### Polybromide determination by Raman analysis

By means of Raman spectroscopy on the electrolyte samples, the occurrence of the different polybromides Br_3_^−^, Br_5_^−^, *etc.* in the prepared 24 samples of HBr/Br_2_/H_2_O (Table 2) is investigated. Electrolyte samples are pipetted into a quartz glass cuvette, sealed and examined under the microscope. The focal point of the Raman beam is positioned just below the edge of the front glass of the cuvette and the position of the carrier table is fixed to obtain reproducible Raman spectra. Surface areas under the polybromide Raman peaks are determined by an integration method according to Levenspiel–Marquart with the Lorentz model. The symmetric and antisymmetric stretching vibrations of Br_3_^−^ and Br_5_^−^ are considered, as well as the symmetric stretching vibration of Br_7_^−^ at fixed Raman shifts known in literature.^41–43^ For the investigation of Br_2_ distribution on the different polybromides, the peaks of symmetric stretching of the polybromides are considered. Areas of the symmetric stretch assigned to the peaks of Br_3_^−^, Br_5_^−^, and Br_7_^−^ are related to the sum of all areas of symmetric stretches to achieve Br_2_ distribution on polybromides *x*(Br_2*n*+1_^−^). Polybromide concentrations *c*(Br_2*n*+1_^−^) are calculated by expecting approximately all bromine *c*(Br_2_)_T_ available in polybromides with the proportion *x*(Br_2*n*+1_^−^) following eqn (7):7
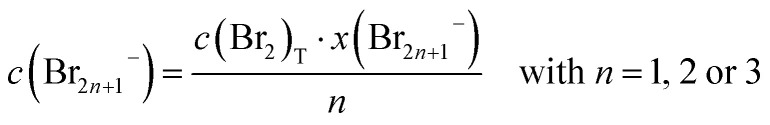


### Simulation of the electrolyte composition

To investigate the validity of polybromide equilibria and equilibrium constants from literature at high reactant concentrations, concentrations of polybromides, HBr and Br_2_ in equilibrium are simulated. To determine total concentrations of Br_2_*c*(Br_2_)_T_, the stoichiometric relationship from eqn (1), including the total HBr concentration *c*(HBr)_T_ and the initial concentration *c*(HBr)_0_ = 7.7 M, is used. The concentrations of polybromides *c*(Br_3_^−^)_eq_, *c*(Br_5_^−^)_eq_, bromine *c*(Br_2_)_eq_, and HBr *c*(HBr)_eq_ in equilibrium are calculated using eqn (4) and (5) with *K*_3_ = 16 L mol^−1^ and *K*_5_ = 37 L^2^ mol^−2^ for each case iteratively including the bromide mass balance eqn (8).

8 *c*(HBr)_eq_ = *c*(HBr)_T_ − *c*(Br^−^_3_)_eq_ − *c*(Br^−^_5_)_eq_

The operating points are defined by the state of charge (SOC) of the electrolyte. The SOC range will be defined in chapter “Definition of the operating range for HBr/Br_2_ electrolytes”. Concentrations are plotted as a function of SOC in chapters “Theoretical concentrations and polybromide equilibria” and “Polybromide distribution and polybromide concentration verification of polybromide equilibria”.

## Results and discussion

### Cell cycling test

**Fig. 3:**
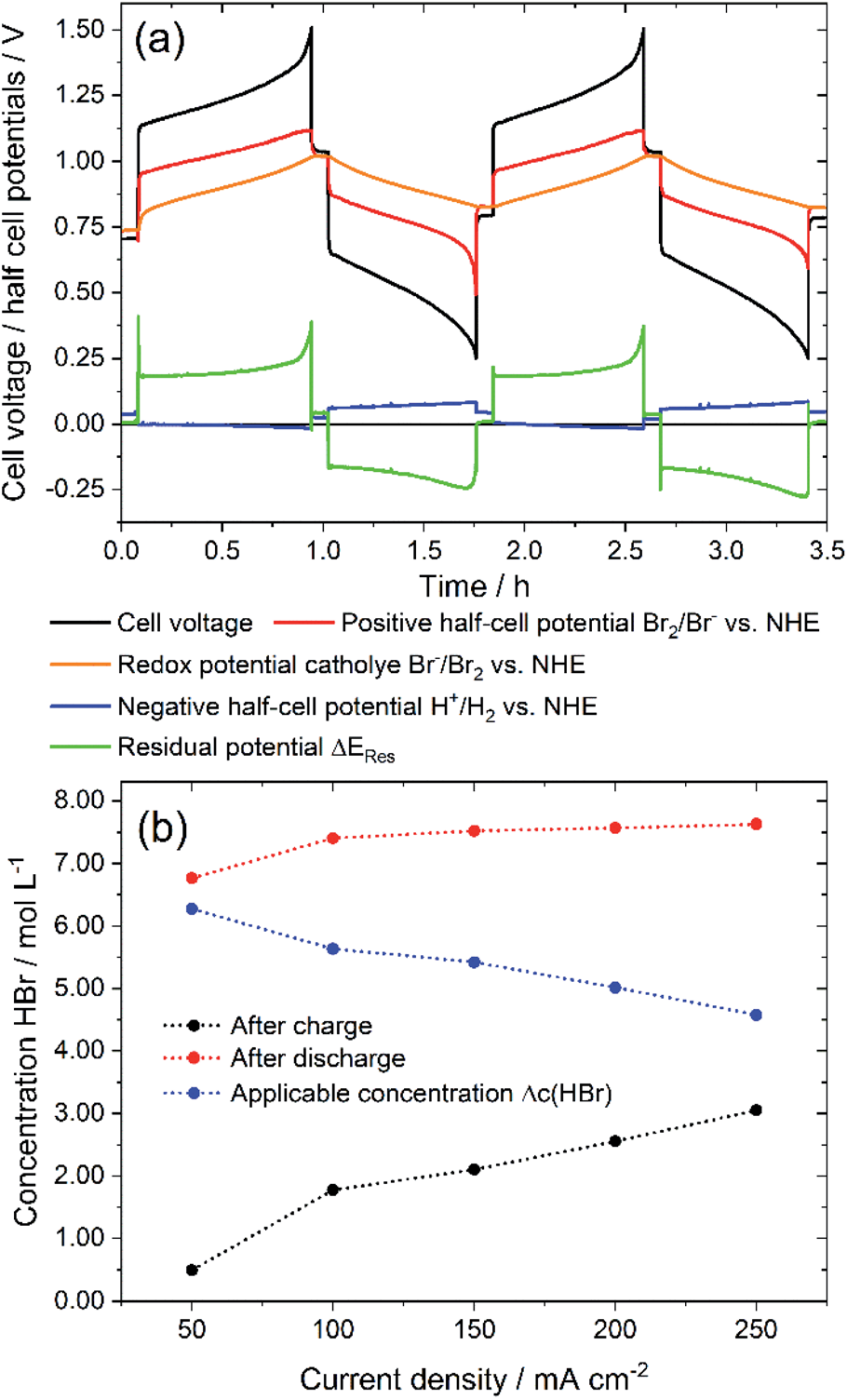
(a) Cell voltage and potentials of a galvanostatic cycling test of a 40 cm^2^ H_2_/Br_2_-RFB single cell with *i* = ±250 mA cm^−2^ depending on operation time: black – cell voltage; red – positive bromine half cell potential, orange – bromine–electrolyte redox potential, blue – negative hydrogen half cell potential, green – residual potential Δ*E*_Res_ according to eqn (6) and (b) maximum charge and discharge concentration of HBr in electrolyte after charge and discharge and applied HBr concentration Δ*c*(HBr) depending on chosen current density.

First, the presented electrolyte is examined in a charge–discharge cell test in a single cell in order to investigate performance characteristics. Voltage and potential curves are qualitatively examined for determining limiting conditions. Cell voltage, half cell potentials and redox potential are shown in Fig. 3a for 2 cycles at a constant charge and discharge current of ±250 mA cm^−2^. A complete charge and discharge of the whole HBr concentration of 7.7 M could not be reached for ±250 mA cm^−2^. Average values of the HBr concentrations after charging and discharging (Fig. 3b) show that a complete charge of 7.7 M HBr to 3.85 M Br_2_ is not achieved. With increasing current density, the useable amount of Δ*c*(HBr) decreases (Fig. 3b). For *i* > 150 mA cm^−2^ there are still *c*(HBr) > 2 M in the solution (Fig. 3b) after charge, while concentrations of HBr after discharge depend less on the current density. Shortly before the end of the two charge cycles the cell voltage rises sharply (Fig. 3a) and at the end of the charging process, individual dark brown drops flow from positive bromine half cell back into the tank. It is believed that a separate Br_2_ phase is formed in the positive half cell and does not get complexed by HBr. The half cell potential of the positive Br_2_/Br^−^ half cell remains constant and does not follow the cell voltage. Instead, it remains at a constant difference from the redox potential. Also, the potential of the negative hydrogen half cell stays constant. As a result of the behaviour of the potentials, mass transport limitation by *c*(HBr) in the positive or negative half cell at the end of the charge process is not present. The introduced residual potential Δ*E*_Res_, which primarily indicates ohmic resistances in the cell, also increases sharply in this area. It is assumed that low conductive bromine is partially accumulated on the surface of the electrode felt and in front of the membrane.

At the end of the discharge process in both cycles the limiting process is the mass transport limitation of bromine in the felt electrode (Fig. 3a). The bromine concentration decreases during the discharge process and would theoretically be 0 M Br_2_ at SOC = 0%. After discharge *c*(HBr) reaches values of 7.45 M up to 7.62 M HBr, which is close to the original concentration of 7.7 M HBr. For all current densities similar effects are recognized.

The main limiting factor for cell performance seems to be the formation of pure Br_2_ leading to higher overvoltage and reducing the electrolyte capacity. The solubility of bromine and the conductivity of the aqueous electrolyte is considered further.

### Electrolyte mixtures and miscibility gap

**Fig. 4:**
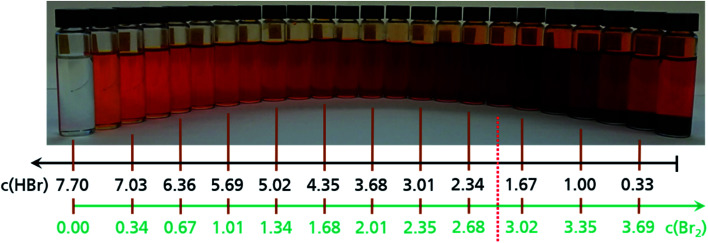
24 glass vials with different HBr/Br_2_ mixtures in aqueous solutions, as used as posolyte in the positive half cell of the H_2_/Br_2_-RFB. Concentration changes can be recognized by the numbered bars. The red dotted line shows the transition between the single-phase aqueous electrolyte (left side) and the two-phase electrolyte system (right side). To the right of the red line for *c*(HBr) ≤ 1.67 M and *c*(Br_2_) ≥ 3.01 M bromine is partially separated and is present parallel to the aqueous phase.

Based on the observation of dark drops emerging from the positive half cell at the end of the charge process, the miscibility of Br_2_ with HBr solutions is investigated *ex situ* for the theoretical concentrations existing in the cell (Fig. 4). The prepared 24 electrolyte samples represent the entire concentration range (Table 2). They show a colour variation due to an increasing bromine concentration from a transparent 7.7 M HBr sample to a deep brown sample at approximately *c*(HBr) = 1.67 M and *c*(Br_2_) = 3.01 M (Fig. 4).

For values *c*(HBr) ≤ 1.67 M and *c*(Br_2_) ≥ 3.01 M a separation of the aqueous electrolyte into two liquid phases is visible. The brown colour intensity of the bromine in the aqueous solution decreases, which indicates a decreasing bromine concentration and is shown in Fig. 4 (right side). The second heavy phase is Br_2_. Samples of the heavy phase are examined by Raman spectroscopy. The characteristic Raman shift is known in literature as a signal for pure bromine.^32,41,44,45^ The samples show a single sharp peak at a Raman shift of *ṽ* = 311 cm^−1^ (Fig. S2, ESI†). In the range between *ṽ* = 1600 cm^−1^ and *ṽ* = 3000–3700 cm^−1^ no further peaks are determined, which would characterise the existence of water. That implies the second liquid phase to be anhydrous, pure bromine Br_2_. If the bromide concentration in the aqueous phase decrease, while the bromine concentration increases, fewer bromide ions are available to form polybromides. Br_2_ is no longer soluble in the aqueous phase and forms a non-miscible second phase consisting of pure Br_2_.

In the bromine half cell of the test cell, performance is limited by the formation of poorly conductive bromine phase even at concentrations of *c*(HBr) that are slightly higher than 1.67 M HBr. Elemental Br_2_ acts as an insulator with a conductivity of 9.6 × 10^−10^ S cm^−1^ (*θ* = 0 °C)^46^ and thus reduces electrolyte conductivity operation ranges. A voltage increase in galvanostatic operation is the result. Due to the oxidation reaction in the bromine half cell during charge, higher local bromine amounts are present than in the tank. In the cell test, a formation of elemental Br_2_ could be calculated already to be present at +250 mA cm^−2^ at concentrations *c*(HBr) ≤ 2.005 M and *c*(Br_2_) ≥ 2.846 M in the cell. Depending on the current density, however, cell charging down to *c*(HBr) < 1 M is possible.

### Solubility curve and empirical representation

To evaluate the exact overlap between the stoichiometrically changing concentration ratios in the H_2_/Br_2_-RFB and the solubility limit, solubility values for Br_2_ in HBr solutions are determined and compared to the working curve of the cell according to eqn (9):9
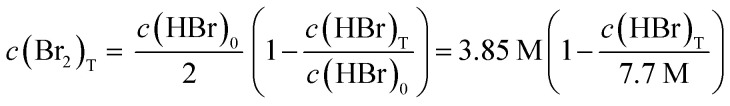


In pure water bromine solubility is 0.22 mol L^−1^, which is approximately in agreement with values from literature.^20–22,47^

**Fig. 5:**
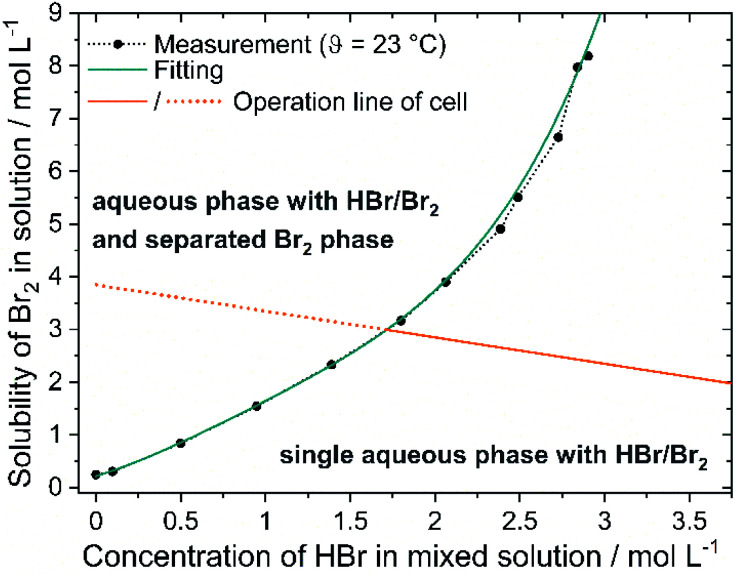
Solubility of Br_2_*c*(Br_2_)_sat_ in aqueous HBr solutions *c*(HBr)_sat_ as a function of the HBr concentration in the solution (black dots and green fitting line) and superposition with the operation line (orange line) of the H_2_/Br_2_-RFB electrolyte by stoichiometric concentration change.

The solubility of bromine in HBr rises with higher HBr concentration due to polybromide formation and is depicted in Fig. 5 (black dots). For saturation concentrations at *c*(HBr)_sat_ < 1.5 M the bromine concentration increases approximately linearly with the bromide concentration. The solubility of bromine increases with increasing bromide concentration for *c*(HBr)_sat_ > 1.5 M. More bromine molecules per bromide ion can be dissolved. For *c*(HBr) < 1.5 M, a ratio < 1.54 between *c*(Br_2_) and *c*(HBr) is calculated. In this range, bromine is theoretically present at approximately 50% as Br_3_^−^ and 50% as Br_5_^−^. For *c*(HBr) ≥ 2.31 M this ratio between complexed bromine and bromide is ≥2 and increases with increasing bromide concentration. Theoretically, only Br_5_^−^ and Br_7_^−^ are present at higher concentrations. Solubility of Br_2_ in HBr was investigated before in literature,^48^ but based on the initial HBr concentration of the experiment. In the H_2_/Br_2_-RFB, saturated concentrations of HBr are present. In this work, however, the concentrations are corrected for mass and volume change and show the bromine concentration in relation to the HBr concentration in the saturated state. The correction is necessary to obtain the solubility of electrolytes in the cell. In order to describe the solubility of bromine in aqueous solutions of HBr empirically for saturated solutions with *c*(Br_2_)_sat_ and *c*(HBr)_sat_, a regression is performed using a 4th degree polynomial function:

10 *c*(Br_2_)_sat._ = *e*·*c*(HBr)_sat._^4^ + *d*·*c*(HBr)_sat._^3^ + *c*·*c*(HBr)_sat._^2^ + *b*·*c*(HBr)_sat._ + *a*

The constants have the following values: *a* = 0.2206 mol L^−1^, *b* = 0.954, *c* = 0.8807 L mol^−1^, *d* = −0.5831 L^2^ mol^−2^ and *e* = 0.1723 L^3^ mol^−3^ and are valid at *θ* = 23 °C between 0 M ≤ *c*(HBr)_sat._ ≤ 2.9 M. The point of intersection between curves representing eqn (9) and (10) is thus the critical point as a solution saturated with bromine is present in the positive half cell depicted by intersection of the green and orange line in Fig. 5: 1.7 M HBr and 3 M Br_2._ For higher concentrations of bromine and lower concentrations of bromide, a second phase is present in the electrolyte mixture and the bromine concentration in the aqueous phase is defined according to eqn (10).

### Conductivity of electrolyte solutions

**Fig. 6:**
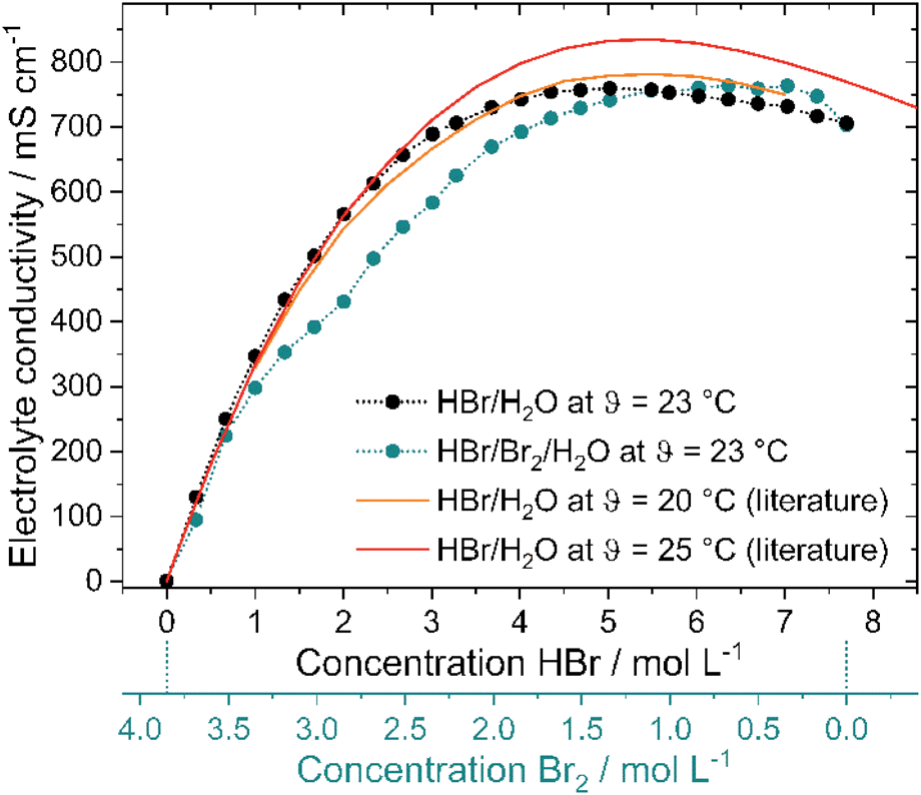
(a) Electrolyte conductivities as a function of the concentration of HBr *c*(HBr) in a pure HBr solution and HBr/Br_2_ solution for a bromine half cell. Measurements shown as points and literature values as lines [11,53] and (b) quantitative ratio between water H_2_O and HBr in the investigated electrolyte solutions as a function of the HBr concentration.

As high current densities are expected due to fast electrode kinetics^7^ and large reaction surfaces in felt electrodes, high electrolyte conductivity throughout the whole operation range is a necessary property of H_2_/Br_2_-RFB electrolytes. Conductivities of pure HBr solutions are known in literature^19,49^ and shall be compared with bromine containing electrolytes and their influence on cell operation. Both depicted lines (Fig. 6 – red and orange lines) in the graph represent conductivity values from available literature^19,49^ at *θ* = 20 °C and 25 °C. With increasing HBr concentration, the measured electrolytic conductivity of both electrolytes at *θ* = 23 °C increases to peak at a certain HBr concentration and slowly declines at high values. Measured conductivities on pure HBr solutions (Fig. 6 – black dots) as well as on HBr/Br_2_ electrolytes (Fig. 6 – green dots) indicate this trend. The measured conductivity of the HBr solutions and the reference curve^49^ at *θ* = 20 °C are in general agreement. For solutions including Br_2_, however, the conductivity increases more slowly. Conductivities are smaller for the application of Br_2_ in the electrolyte for *c*(HBr) < 5.69 M compared to pure HBr/H_2_O electrolyte. For *c*(HBr) ≥ 1 M (or *c*(Br_2_) ≤ 3.35 M) in both electrolytes high conductivities of *κ* ≥ 298 mS cm^−1^ are relevant for the operation of the electrolytes in the battery. In parallel or *c*(HBr) < 1 M or *c*(Br_2_) > 3.35 M the electrolytic conductivity decreases sharply to *c*(HBr) = 0 M/*c*(Br_2_) = 3.85 M with conductivity close to the one of pure water. In this range the bromine remains predominantly in its own separate phase and has little influence on the conductivity of the aqueous solution.

High conductivities up to 763.2 mS cm^−1^ are achieved by the transport of charges by means of the Grotthus mechanism in acidic aqueous solutions.^50,51^ Conductivity peaks of both electrolytes (*c*(HBr) = 5.02 M, *κ* = 759.3 mS cm^−1^ in HBr/H_2_O electrolytes and *c*(HBr) = 6.36 M, *κ* = 763.2 mS cm^−1^, in HBr/Br_2_/H_2_O electrolytes) depend on the dissociation of HBr. For low HBr concentrations it is assumed that hydrobromic acid with p*K*_s_ = −9^18^ is completely dissociated. Due to the formation of a solvate shell around the oppositely charged ions, ions are protected to form strong ionic interactions with each other. For higher HBr concentrations in the range of the peaks, the degree of dissociation is limited (70% at *c*(HBr) = 6.27 M).^52^ The quantity of water molecules is no longer sufficient in relation to the HBr molecules present to form a solvate shell for all bromide ions and protons. Oppositely charged ions attract each other and cannot move freely in the electric field due to the lack of a solvation shell. The Grotthus mechanism is inhibited. This state predominates in the peak range and for higher concentrations in bromine-containing electrolytes.

For *c*(HBr) > 5.69 M and *c*(Br_2_) < 1 M (752.8 mS cm^−1^ at 5.59 M HBr) the conductivity of the bromine-containing electrolytes is higher than the conductivity of the bromine-free electrolytes. As different polybromides in low amounts are present in the bromine-containing solution, a regular order of weakly solvated protons and bromide ions in the high concentrated HBr solution is not available anymore. Polybromides have larger hydrodynamic radii, including lower local charge densities. Protons and bromide ions attract each other less strongly. This results in irregularities in the arrangement of protons and bromide ions. The Grotthus mechanism can proceed more simply as the required oxonium ions and water molecules are bound less rigidly and in an orderly manner to a fixed place in the solution. However, availability of Br_2_ in the range of *c*(HBr) > 5.69 M has minor influence on the general high conductivity of those electrolytes.

The influence of polybromides on conductivity is described in the last subchapter whereas the polybromide composition of the electrolytes is discussed in the chapter Raman analysis of the electrolyte samples.

### Definition of the operating range for HBr/Br_2_ electrolytes

The definition of the operation range in terms of limiting concentrations is defined by the state of charge range (SOC). It is determined here on the basis of conductivity and the miscibility gap. SOC = 0% refers to the electrolyte mixture of 7.7 M HBr/0 M Br_2_ in H_2_O, with a conductivity of *κ* = 705.8 mS cm^−1^. For SOC = 100% the operation point with 1 M HBr/3.35 M Br_2_ in aqueous solution is chosen. Charging the electrolyte below *c*(HBr) < 1 M is not convenient due to the sharp drop in electrolytic conductivity *κ* < 298.0 mS cm^−1^. In addition, from SOC > 89.40%, the working range of the positive bromine half cell is superimposed with the limited solubility of Br_2_ in the aqueous electrolyte. Although the formation of bromine in the positive half cell leads to ohmic overvoltage, it strongly depends on the electrode type, as well as on the current density and flow rate. A small overlap of the operation curve and the formation of elemental bromine is acceptable in order to achieve a high usable electrolyte capacity at the same time. The total concentrations of HBr and Br_2_ as a function of SOC are given by eqn (11) and (12).

11 *c*(HBr)_T_ = 6.7 M(1 − SOC) + 1 M

12 *c*(Br_2_)_T_ = 3.35 M·SOC

Concentrations of HBr and Br_2_ as a function of SOC are shown in Table S1 (ESI†).

In some of the following diagrams, SOC values of up to 115% are shown. In this range concentration values of *c*(HBr) < 1 M HBr are given. SOC = 115% corresponds to 0 M HBr/3.85 M Br_2_. Thus, the entire concentration range between 0 and 7.7 M HBr and 0 and 3.85 M Br_2_ is shown. With the definition of SOC, a utilizable concentration Δ*c*(HBr) = 6.7 M is achievable, which corresponds to a capacity of 179.57 A h L^−1^ of the liquid electrolyte.

### Theoretical concentrations and polybromide equilibria

**Fig. 7:**
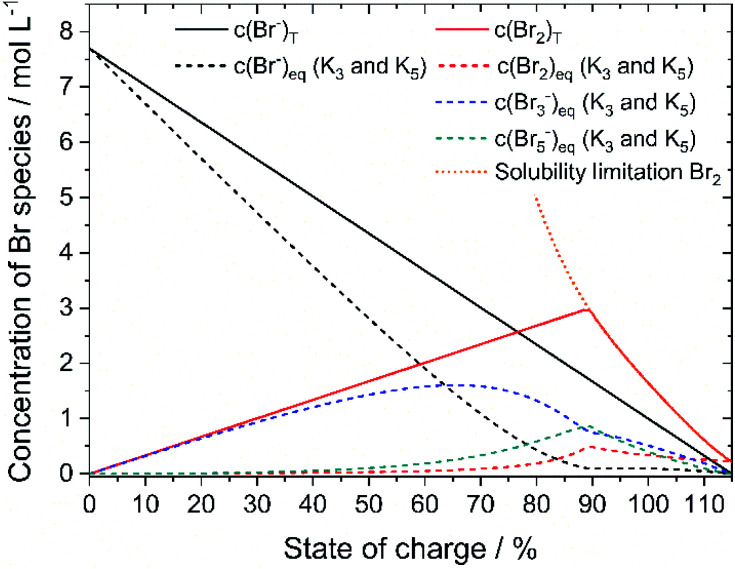
Plot of the theoretical concentration of HBr_T_, Br_2,T_, Br_3,eq_^−^, Br_5,eq_^−^ with *K*_3_ = 16 L mol^−1^ and *K*_5_ = 37 L^2^ mol^−2^ including bromine solubility limit.

Based on the definition of the SOC range the total concentrations of bromine *c*(Br_2_)_T_ and HBr *c*(HBr)_T_ are calculated following eqn (11) and (12) and considering the solubility limitation according to eqn (10). Including polybromide equilibria for Br_3_^−^ and Br_5_^−^, their concentrations at equilibrium are calculated for different SOCs applying polybromide law of mass action eqn (4) and eqn (5). Values of *K*_3_ = 16 L mol^−1^ and *K*_5_ = 37 L^2^ mol^−2^ are applied for the entire SOC range. Based on these values also bromine concentration *c*(Br_2_)_eq_ and HBr concentration *c*(HBr)_eq_ at equilibrium are considered. Simulated concentrations are shown in Fig. 7.

Since *K*_3_ and *K*_5_ > 1, both equilibria shift the reaction to form polybromides. Due to the strong excess of bromide Br^−^ for small SOCs and the low concentrations of Br_2_, almost the complete amount of Br_2_ is converted to Br_3_^−^ for SOC < 60% (Fig. 7). While *c*(HBr)_eq_ decreases, and *c*(Br_2_)_T_ increases in total, more Br_5_^−^ is formed. A maximum peak for *c*(Br_3_^−^) at SOC = 65.51% and a peak for Br_5_^−^ at SOC = 89.4% appear during SOC increase in Fig. 7. Their location is related mainly on the equilibria based on the law of mass action according to eqn (4) and (5), where the bromine concentration is proportional and quadratic respectively, and the comparison of the equilibrium constants is *K*_3_ < *K*_5_. The existing concentration of free bromide ions Br^−^ is lower than the global concentration as a part of the bromide is bound in the polybromides. The amount of free bromine *c*(Br_2_)_eq_ is small compared to polybromide concentrations. Heptabromides are not included in the simulation because its equilibrium constant is not known in the literature. Taking the solubility limit of Br_2_ in aqueous HBr solutions according to eqn (10) into account, the concentration of Br_2_ in aqueous HBr solutions decreases for SOC > 89.40% (*c*(HBr) < 1.7 M and *c*(Br_2_) > 3.0 M) (Fig. 7). As a result, less Br_5_^−^ is formed and more Br_3_^−^ is present over a wider SOC range. The bromine concentration *c*(Br_2_)_eq_ slowly decreases to the measured solubility limit of 0.22 mol L^−1^ Br_2_ in water at SOC = 115%. Values in Fig. 7 are used to compare simulated values with real measured polybromide concentrations.

### Raman analysis of the electrolyte samples

**Fig. 8:**
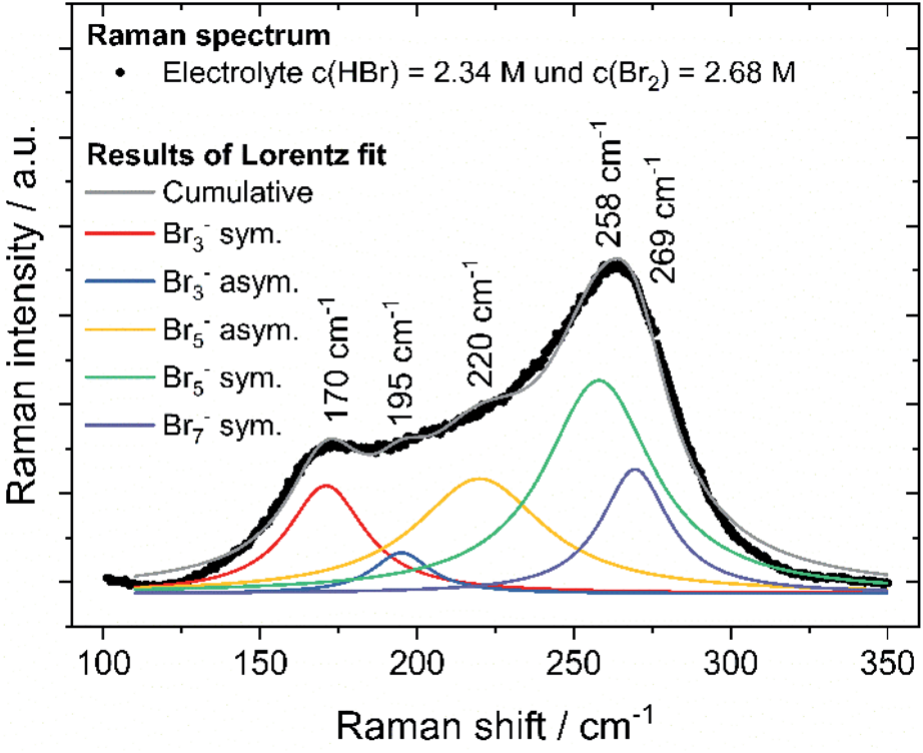
Raman spectrum of the electrolyte mixture with *c*(HBr) = 2.34 M and *c*(Br_2_) = 2.68 M (SOC = 80%) and water including Raman fitting for the symmetrical and antisymmetrical stretching oscillation of tribromide, pentabromide and heptabromide.

To prove the validity of the assumed polybromide equilibria, polybromide concentrations are determined experimentally. To evaluate the polybromide composition of the 24 aqueous solutions, electrolyte samples are examined by Raman spectroscopy and show Raman peaks in the range of the Raman shift between ṽ = 125 cm^−1^ and 325 cm^−1^, shown in example for SOC = 80% in Fig. 8. Raman spectra of all 24 investigated samples are shown in the ESI (Fig. S1–S5†). Two main peaks at *ṽ* = 170 cm^−1^ and *ṽ* = 260 cm^−1^ are prominent in the spectra, which are mainly assigned to symmetrical stretching vibration of Br_3_^−^ (*ṽ* = 170 cm^−1^) and Br_5_^−^ superimposed by Br_7_^−^ (*ṽ* = 260 cm^−1^). Due to the increasing Br_2_ content over the SOC range, Br_5_^−^ peak at *ṽ* = 258 cm^−1^ first increases. For SOC > 60%, bromine is increasingly stored in Br_7_^−^ at the expense of Br_5_^−^. The Br_5_^−^ concentration decreases while the Br_7_^−^ concentration increases, which leads to a shift of the main peak to higher Raman shifts at *ṽ* = 269 cm^−1^ (Fig. S1 and S2 in ESI†). The presence of Br_7_^−^ as higher polybromide at high bromine concentrations is observed for those electrolytes. An equilibrium of Br_7_^−^ is needed to describe the existence of Br_7_^−^:

13 Br^−^ + 3Br_2_ ⇌ Br_7_^−^

For the samples with SOC > 100%, an additional weak shoulder overlapping the strong signal of the Br_7_^−^ is visible. This peak is assigned to dissolved elemental Br_2_ at *ṽ*(Br_2_)sym. = 311 cm^−1^ and is found only when the phases of bromine and electrolyte separate. The peak is weak, indicating only minor amounts of elemental Br_2_ present and too weak to be fitted. Bromine, which cannot pass into solution as polybromide, is present in small amounts in aqueous electrolyte there.

Br_2_ distribution on the different polybromides *x*(Br_2*n*+1_^−^) was calculated from the strongly responding peaks of symmetric stretching of the polybromides only, after fitting and shown in Fig. 8. All fits achieve a coefficient of determination *R*^2^ of 0.975 or higher and are shown in the ESI in Fig. S3 to S5.† Due to this method, polybromide concentrations in aqueous solution are determined and their influence on other performance parameters is investigated. Also, the existence of polybromides available in high concentrated bromine electrolyte solutions is proven.

### Polybromide distribution and polybromide concentration verification of polybromide equilibria

**Fig. 9:**
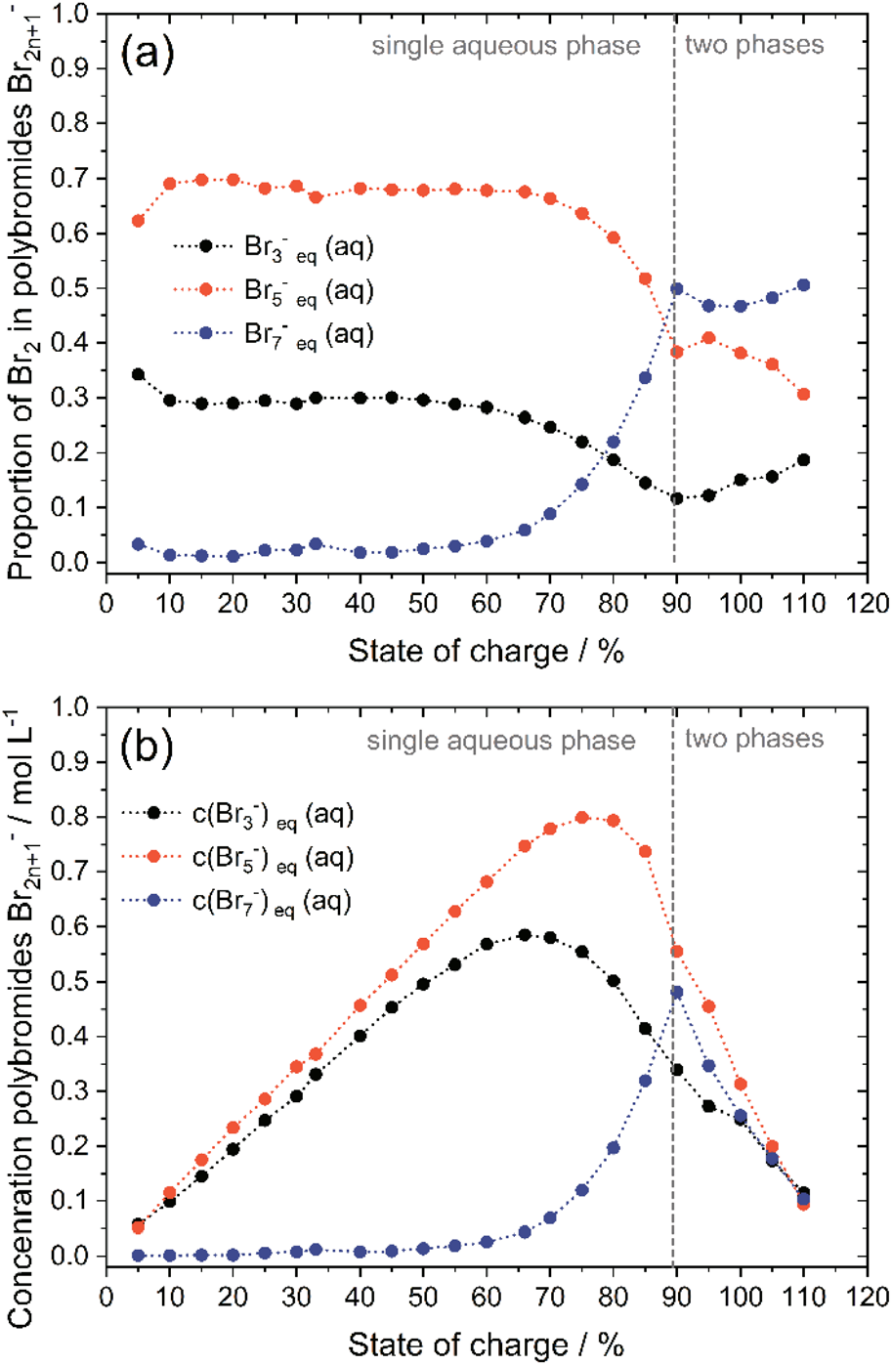
(a) Distribution of bromine on different polybromides *x*(Br_3_^−^), *x*(Br_5_^−^) and *x*(Br_7_^−^) for the investigated electrolyte mixture as a function of SOC and (b) polybromide concentrations as a function of SOC for the investigated electrolytes.

To validate the accuracy of the simulated polybromide equilibria and concentrations and the applicability of their equilibrium constants, the distribution of Br_2_ across the three polybromide types and the concentrations of the polybromides are shown in Fig. 9. Br_3_^−^ and Br_5_^−^ are present throughout the entire SOC range (Fig. 9a), while the distribution of Br_2_ in Br_5_^−^ predominates. Br_7_^−^ is available in small amounts for SOC < 60% and rises for higher SOC values. The proportions of bromine stored in Br_3_^−^, Br_5_^−^ and Br_7_^−^ are almost constant for low SOC values with *x*(Br_3_^−^) = 0.288 (SOC ≤ 45%), *x*(Br_5_^−^) = 0.695 (SOC ≤ 66%), *x*(Br_7_^−^) = 0.011 (SOC ≤ 45%) and almost independent of the SOC (Fig. 9a). There is no dependence of the polybromide distribution on the SOC and therefore also on the total bromine concentration *c*(Br_2_)_T_ as well as the concentrations of free bromide ions *c*(HBr)_eq_. For SOC ≤ 45%, the bromine distribution leads to an approximately linear increase in the concentration of polybromide ions as a function of *c*(Br_2_)_T_ (Fig. 9b). For SOC > 45% and 66% the proportion of Br_2_ in Br_3_^−^ and Br_5_^−^, respectively, are decreasing till reaching the solubility limit at SOC = 89.40%, while the proportion of Br_2_ in Br_7_^−^ is rising continuously within that range. All values for distribution of Br_2_ on polybromides and polybromide concentrations are listed in Table S2 in the ESI.†

**Fig. 10:**
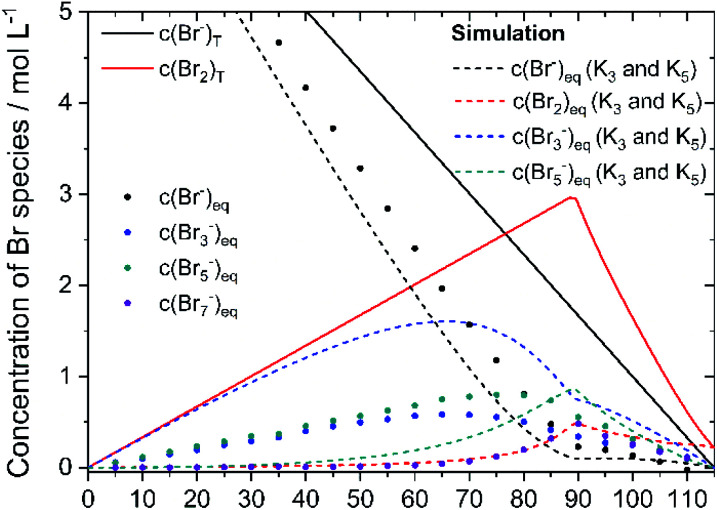
Comparison of polybromide concentrations from measurements (dots) with simulated values based on polybromide equilibria from the literature (dashed line) next to global and equilibrium concentrations of bromine (red – line and dashed line) and HBr (black – line, dots and dashed line).

To verify the presence of the polybromide equilibria according to eqn (2) and (3) and the applicability of the equilibrium constants to the polybromide concentrations, the measured and calculated polybromide concentrations are compared in Fig. 10. The measured polybromide concentrations do not correspond to the simulated values in Fig. 10. Over the whole SOC range much lower concentrations of Br_3_^−^ are measured than calculated in the simulation. In parallel measured Br_5_^−^ concentrations are higher than calculated values in the simulation. One reason for this discrepancy can be sought in the presence of Br_5_^−^ from SOC = 0%, although eqn (5) with *K*_5_ = 37 L^2^ mol^−2^ and eqn (4) predict an occurrence of Br_5_^−^ only at high total Br_2_ concentrations. According to eqn (5), therefore, much higher values for *K*_3_ and *K*_5_ need to be expected for these concentrations, or the equilibrium model according to eqn (3) is not valid in the chosen concentration range. From SOC > 66%, the Br_7_^−^ content is relevant and increasing. As Br_7_^−^ incorporates three times the amount of bromine molecules compared to Br_3_^−^, the amount of Br_3_^−^ and Br_5_^−^ decreases further (Fig. 9b). Related to the differences between simulation and measurement and the constant bromine distribution according to Fig. 9a for SOC < 45%, it is assumed that in addition the equilibrium concentrations in these non-ideal solutions are strongly dependent on the electrolyte composition.

The equilibrium constants *K*_3_ = 16 L mol^−1^ and *K*_5_ = 37 L^2^ mol^−2^ and the associated equilibria eqn (2) and (3) do not adequately describe the distribution of Br_2_ for these selected electrolyte concentrations as a function of the SOC. The Br_7_^−^ equilibrium must be considered in addition.

### Discussion of polybromide distribution in aqueous electrolytes

In order to understand the different involved phenomena, the SOC range is separated in three parts: part 1 from 0 ≤ SOC ≤ 45%, part 2 from 45% < SOC ≤ 89.40% and part 3 for SOC > 89.40%.

In the area SOC ≤ 45% (part 1) a sufficiently excess quantity of HBr is present in the solution (Fig. 10) and all converted bromine molecules stay in solution as respective polybromides, predominantly Br_3_^−^ and Br_5_^−^. Free bromide *c*(HBr)_eq_ is still available in excess. Linearly rising concentrations of polybromide concentrations in Fig. 9b are a consequence of constant Br_2_ distribution on the three different polybromides and linearly rising total Br_2_ concentration with the SOC. Although the measured concentrations of Br_3_^−^ are lower in this range than the simulated concentrations for Br_3_^−^, for SOC < 80% the concentrations of simulated and measured values are directly proportional. This is an indication that the tribromide equilibrium according to eqn (2) is valid. The directly proportional relationship between the concentrations of Br_3_^−^, Br_5_^−^ and Br_7_^−^, however, indicates a direct relationship between the polybromides. In the area for SOC ≤ 45% a sequential equilibrium order is suggested with the original Br_3_^−^ equilibrium according to eqn (2), and the following equilibria according to eqn (14) and (15):

14 Br_3_^−^(aq) + Br_2_ ⇌ Br_5_^−^(aq)

15 Br_5_^−^(aq) + Br_2_ ⇌ Br_7_^−^(aq)

Duranti *et al.*^53^ also applies sequential storage of Br_2_ up to Br_5_^−^ following eqn (2) and (14), but did not justify his assumption experimentally.

It seems that bromine molecules which are not bound in polybromides form a separate phase and probably are not included in the mass action law in these areas. As a consequence, they are considered in the law of mass action leading to a formal activity of bromine of 1. However, it is found that the ratio between *c*(Br_3_^−^) and *c*(Br_5_^−^) between 0% ≤ SOC ≤ 60% is almost constant, why eqn (5) is replaced by eqn (16):16
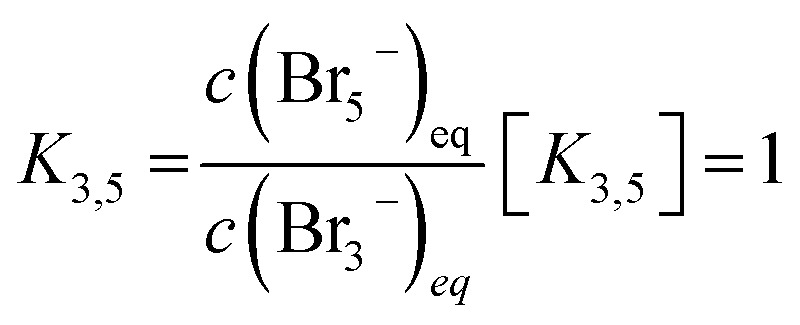


For Br_7_^−^ the same linear relation to Br_5_^−^ is suggested:17
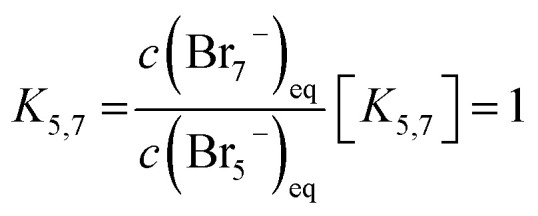


In the range for 0% ≤ SOC ≤ 45% the equilibrium constants are described with the following values *K*_3_ ≈ 4.734 L mol^−1^, *K*_3,5_ = 1.163 and *K*_5,7_ = 1.89 × 10^−2^.

For 50% < SOC ≤ 89.40% (part 2) the concentrations of Br_3_^−^ (SOC ≥ 50%) and Br_5_^−^ (SOC ≥ 70%) increase at a slower rate (Fig. 9b). Both concentration curves reach a peak point and then decrease with increasing SOC. For Br_3_^−^ the peak point is at SOC = 65% with *c*(Br_3_^−^) = 0.585 M and for Br_5_^−^ at SOC = 75% with *c*(Br_5_^−^) = 0.800 M. The concentration of Br_7_^−^ rises progressively in this range. At SOC = 90%, the concentration peak of Br_7_^−^ results at *c*(Br_7_^−^) = 0.481 M. The tendency to form higher polybromides increases with increasing total bromine concentration *c*(Br_2_)_T_ and simultaneously decreasing bromide concentration *c*(HBr)_eq_ starting from SOC ≥ 50%. The amount of free HBr in solution is not sufficient to take up all the Br_2_. Less Br_3_^−^, but more Br_7_^−^ is formed. For SOC ≥ 66% Br_2_ is increasingly stored by further conversion of the Br_3_^−^ to Br_5_^−^ and to Br_7_^−^. For SOC ≥ 75% and above, Br_5_^−^ and Br_3_^−^ concentrations decrease further by the uptake and formation of Br_7_^−^. But joint application of equilibria following eqn (2), (3) and (13) is not valid so far in this concentration range. A mathematical relation for *K* values in this range has not been found.

In part 3 for SOC > 89.4% the solubility of bromine is limited following eqn (10). The dominance of Br_7_^−^ compared to Br_3_^−^ can be seen in the distribution of Br_2_ on the polybromides (Fig. 9a). For SOC > 60% the amount of Br_2_ in Br_7_^−^ increases strongly, while the amounts of Br_2_ in Br_5_^−^ and Br_3_^−^ decrease. Crossing the solubility limit for SOC > 89.40%, the amount of bromine in Br_3_^−^ increases slowly with rising SOC from high to low concentrations of HBr. Jones and Beackström^20^ tried to explain this effect in 1934 theoretically from the solubility measurements of Br_2_ in KBr solutions, without having access to Raman spectroscopy. They discussed that the formation of Br_3_^−^ is not sufficient describing the rising solubility of Br_2_ in aqueous solutions, and stated the existence of Br_5_^−^.^20^ It can even be concluded that with increasing Br^−^ concentration the tendency to form Br_7_^−^ is pronounced and most proportion of Br_2_ is stored in Br_7_^−^.

### Influence of polybromides on the conductivity

As the polybromide distribution and concentrations are known for the entire SOC range, it is necessary to step back and evaluate their dependence on the conductivity (Fig. 6 – green dots). Between 2.0 M < *c*(HBr) < 5.69 M, the conductivity of the bromine-containing solution is lower than that of the bromine-free series. The presence of bromine and especially polybromides reduces the conductivity. The difference between pure HBr/H_2_O solutions and HBr/Br_2_/H_2_O solutions is most significant at *c*(HBr) = 2 M/*c*(Br_2_) = 2.85 M with Δ*κ* = 132 mS cm^−1^, with the highest bromine and Br_7_^−^ concentration in the aqueous solution. The most relevant is the hinderance of polybromides on the Grotthus mechanism in solution with the oxonium ions and water molecules. Due to the larger molecule volumes of the hydrolysed polybromides and the increasing concentration of polybromides in aqueous solution, the charges can no longer be transferred in the direct way, but must bypass the larger polybromide ions. Next to Grotthus charge transport slower migration of polybromides in the electric field is expected. For higher polybromides, a larger hydrodynamic radii are expected. The equivalent conductivities of bromide and polybromides in literature with *λ*(Br^−^) = 78.1 S cm^2^ mol^−1^;^19,34,54^*λ*(Br_3_^−^) = 43 S cm^2^ mol^−1^;^20^*λ*(Br_5_^−^) = 15.6 S cm^2^ mol^−1^ (ref. 54) or *λ*(Br_5_^−^) = 30 S cm^2^ mol^−1^ (ref. 20) show the corresponding trend. No equivalent conductivities for Br_7_^−^ are available in the literature.

It can be concluded that higher polybromides like Br_5_^−^ or Br_7_^−^ lead to lower electrolyte conductivities, but their formation is related to high bromine concentration *versus* low bromide concentration available in solution. In general, conductivities are high as Grotthus mechanism of protons in water dominates the transport of charges in the electrolyte.

## Conclusions

The electrolyte composition and the operation limits of a high capacity bromine electrolyte for a H_2_/Br_2_-RFB were investigated in detail based on 48 wt% HBr solution, bromine and water. The most favourable operation range was defined based on the results. For discharged electrolyte at SOC = 0% a concentration of 7.7 M HBr and 0 M Br_2_ in H_2_O and for charged electrolyte at SOC = 100% of 1 M HBr and 3.35 M Br_2_ were defined. Future work will focus on the application of additives in this electrolyte, which are added to the electrolyte and are intended to bind Br_2_. The addition of these additives leads to a dilution of the commercially available hydrobromic acid HBr 48 wt%. Therefore, for SOC = 0% a HBr concentration of 7.7 M is introduced. The definition of concentrations at SOC = 100% is mainly based on two effects: (1) the solubility of bromine in electrolytes with *c*(HBr) < 1.7 M and *c*(Br_2_) > 3 M is limited and leads to a formation of non-conductive pure Br_2_ phase in the H_2_/Br_2_-RFB. In parallel (2) high electrolyte conductivities in the entire SOC range are achieved between 298 ≤ *κ* ≤ 763 mS cm^−1^ (*θ* = 23 °C). Polybromides in solution slightly lower the conductivity compared to pure HBr/H_2_O solutions. Due to the chosen high concentrated HBr/Br_2_ electrolytes in combination with the limitations a high electrolyte capacity of 179.6 A h L^−1^ is achieved.

It was found that bromine is present in aqueous solution in the form of not only Br_3_^−^, Br_5_^−^, but also most probably Br_7_^−^. Since polybromides are not present individually in aqueous solution, Raman spectroscopy was used to determine the polybromide proportions in solution. The distribution of bromine in Br_3_^−^, Br_5_^−^ and Br_7_^−^ is largely constant and independent of the HBr and Br_2_ concentrations for SOC ≤ 45%. For SOC ≥ 50% there is a lack of HBr compared to Br_2_, so that higher polybromides such as Br_7_^−^ are formed at the expenses of lower polybromides such as Br_3_^−^ and Br_5_^−^. In addition, higher polybromides in form of Br_7_^−^ need to be considered to describe the state of the electrolyte. A verification of the chemical polybromide equilibria known in the literature shows that for high concentration of >1 M HBr and Br_2_ the concentration-based mass action laws and their equilibrium constants proposed in the literature cannot be applied. For SOC < 45%, a proposal for existing equilibria and equilibrium constants was developed.

## Credit author statement

Michael Küttinger: conceptualization, methodology, experimental investigation, data analysis, visualisation, simulation, writing – original draft, writing – editing and review. Jakub K. Wlodarczyk: data analysis, visualisation, writing–editing and review. Daniela Daubner: methodology, experimental investigation, data analysis. Peter Fischer: writing–editing and review. Jens Tübke: writing–editing and review.

## Conflicts of interest

There are no conflicts to declare.

## Supplementary Material

RA-011-D0RA10721B-s001
