# Supplementary material for: High energy density electrolytes for H2/Br2 redox flow batteries, their polybromide composition and influence on battery cycling limits
Source: RSC Adv. 2021 Jan 28;11(9):5218–29. doi: 10.1039/d0ra10721b (PMC8694680; doi:10.1039/d0ra10721b)
Supplement: RA-011-D0RA10721B-s001 [file RA-011-D0RA10721B-s001.pdf]

Electronic Supplementary Material (ESI) for RSC Advances.
This journal is © The Royal Society of Chemistry 2021

Electronic supplementary information (ESI)

to the manuscript "High energy density electrolytes for H_2/Br_2 redox flow batteries, their polybromide composition and influence on battery cycling limits" by Michael Küttinger, Jakub K. Włodarczyk, Daniela Daubner, Peter Fischer and Jens Tübke.

In the ESI the reader will find detailed information on the measurement methods used, parameter settings for measurements, chemicals and purities as well as evaluation steps. In addition, Raman results on aqueous $HBr/Br_2/H_2O$ electrolytes are shown in figures and tables.

Experimental and methods

Chemicals

All analysed electrolytes are composed of distilled water (PURELAB Ultra Analytic/Veolia-ELGA), hydrobromic acid HBr 48 wt% (Alpha Aesar, 48 wt%) and elemental bromine Br_2 (Alpha Aesar, +98 wt%).

Electrolyte preparation

For this electrolyte investigation an aqueous 7.7 M HBr solution is the initial electrolyte solution. A lower concentration compared to commercially available hydrobromic acid (HBr 48 wt%) with a difference of around 1.1 M is used, to allow the replication of other dilutions. Charging and discharging are theoretically possible between 7.7 M HBr and 0 M HBr. This range is evaluated with 24 samples, with a concentration change of $\Delta c(HBr)$ = -0.335 M and $\Delta c(Br_2)$ = 0.167 M per sample. The correlation between $\Delta c(HBr)$ and $\Delta c(Br_2)$ is stoichiometric and represented by eq. 1. The volume (V) of each sample is V = 10 mL. Concentrations of HBr and Br_2 in solution are shown in table 2 in the main manuscript. Each sample is used for the investigation of (i) electrolytic conductivity of the solution, (ii) detection of a possible miscibility gap of the solutions and (iii) Raman investigations for polybromide detection as a basis for the calculation of polybromide concentrations and equilibrium constants of the polybromide equilibria according to equations 4 and 5 (main manuscript). In addition to $HBr/Br_2/H_2O$ samples described above, samples without Br_2 based on HBr/H_2O are mixed in order to investigate reference conductivities of pure HBr/H_2O electrolytes and compare them with solutions including Br_2. 24 samples are prepared with a volume of V = 10 mL and a concentration change of $\Delta c(HBr)$ = -0.335 M in aqueous solution. For all samples, the amount of water is constant starting from a solution with c(HBr) = 7.7 M corresponding to the theoretical conditions in a positive bromine half-cell of a H_2/Br_2-RFB.
For a cell test, V = 60 mL of discharged electrolyte with c(HBr) = 7.7 M HBr in H_2O was prepared.

Cycling cell test

A cell test is performed with a test cell developed at Fraunhofer ICT. As membrane electrolyte assembly (MEA) a Nafion 117 membrane with single sided Pt/C catalyst loading (3 mg Pt cm^{-2}) from Baltic Fuel Cells (Germany) is used as hydrogen half-cell catalyst. The membrane has a geometrically active surface area of 40 cm². The gas diffusion electrode of the negative hydrogen half-cell consists of a gas diffusion layer (GDL) BC29 from SGL Carbon (Germany) and a current collector FU 4369 from Schunk Kohlenstofftechnik (Germany) with a milled meander structure to supply the gas anode with hydrogen and to remove formed hydrogen and water. The anode side is continuously fed with hydrogen from a gas cylinder with 100 mL min^{-1} at 1.013 bar or higher, leading to stoichiometric factor $\lambda \geq 1.31$. There is 31 % more hydrogen available compared to the amount needed during discharge. The hydrogen produced during the charging process is not stored. No external humidification of the hydrogen is applied during the measurements. The investigation concerns the bromine half-cell only. There, the electrode consists of a GFA 5 graphite felt from SGL Carbon (Germany), embedded in a 3 mm deep frame, which is closed by a glassy carbon (GC) current collector Sigradur G (HTW, Germany). The cell is charged and discharged while a flow rate of 20 mL min^{-1} at different current densities of +/-50 mA cm^{-2}, +/-100 mA cm^{-2}, +/-150 mA cm^{-2}, +/-200 mA cm^{-2} and +/-250 mA cm^{-2} is applied. Voltage thresholds of the experiments are at E_{max} = 1.5 V and E_{min} = 0.25 V. The electrolyte is located in a glass tank in which a GC rod Sigradur G (HTW, Germany) and a mercury sulphate reference electrode (RE) $Hg/Hg_2SO_4/K_2SO_4$(sat.) (SI Analytics, Germany) are installed and in contact with the electrolyte (figure 2 in main

manuscript). On one hand, the redox potential of the catholyte at the GC rod versus $Hg/Hg_2SO_4/K_2SO_4$(sat.) is measured. On the other hand the half-cell potential $\phi(Br_2/Br^-)_{i\neq 0}$ during charge and discharge operation of the bromine half-cell connected via the electrolyte in the tube against $Hg/Hg_2SO_4/K_2SO_4$(sat.) is measured, according to figure 2 in the main manuscript. The negative hydrogen half-cell contains a dynamic hydrogen electrode (DHE) according to [1] to determine the potential of the hydrogen anode. The cell voltage $E_{Cell\, i\neq 0}$ under load, the redox potential $\phi(Br_2/Br^-)_{redox}$ versus a $Hg/Hg_2SO_4/K_2SO_4$(sat.) and half-cell potentials of the positive bromine half-cell $\phi(Br_2/Br^-)_{i\neq 0}$ vs. the normal hydrogen electrode (NHE) and the negative hydrogen half-cell $\phi(H^+/H_2)_{i\neq 0}$ vs. NHE were determined in parallel during cycling experiments. A so-called "residual potential" ΔE_{Res} is calculated from these values according to eq. 1:

$$\Delta E_{Res} = E_{Cell\, i\neq 0} - (\phi(Br_2/Br^-)_{i\neq 0} - \phi(H^+/H_2)_{i\neq 0}) \qquad \text{eq. 1}$$

It essentially reproduces the overvoltages between the anode and cathode, and represents the sum of electrolyte resistance and membrane resistance. Kinetic inhibitions and mass transport limitation at the electrodes are represented by the half-cell potentials. Qualitative noticeable occurrences of cell voltage behaviour are determined from the galvanostatic cycle test of a test cell.

The measurement setup is shown in figure 2 in the manuscript including all voltage and potential measurements.

Investigation of the miscibility

24 electrolyte samples are prepared with the limiting concentrations of 7.7 M HBr/0 M Br_2 and 0 M HBr/3.85 M Br_2. The samples are examined by visual inspection at ϑ = 23 °C for any phase separation of the aqueous phase and a non-soluble bromine phase. The glass vials are observed from below against a light source and drops or a complete coating of the vials' bottom with a non-transparent deep brown bromine phase are used as a criterion for a miscibility gap.

Conductivity of electrolyte solutions

Electrolytic conductivities are measured for all 48 samples between the concentration limits at ϑ = 23°C using an LF1101 conductivity cell (SI Analytics/Germany). Electrolytes with $HBr/Br_2/H_2O$, and electrolytes without Br_2 in solution are examined. Ohmic resistances of the electrolytes $R_{Electrolyte}$ are measured by potentiostatic impedance spectroscopy with an excitation of û = 10 mV (amplitude) in the frequency range between 1 MHz and 100 Hz at a potential offset of 0 mV and converted into electrolytic conductivities κ according to eq. 3. The cell constant $K_{Conductometer}$ of the conductivity cell is determined using 1 M KCl solution and a known conductivity of 303.9 mS cm^{-1} at ϑ = 23 °C (determined by linear regression for ϑ = 23 °C from [2] (eq. 2):

$$K_{Conductometer} = \frac{A}{l} = \frac{1}{R_{1M\,KCl,\ t=23°C} \cdot \kappa_{1M\,KCl,\ t=23°C}} \qquad [K] = cm \qquad \text{eq. 2}$$

$$\kappa_{Elektrolyt,\ t=23°C} = \frac{1}{R_{Electrolyte,\ t=23°C} \cdot K_{Conductometer}} \qquad [\kappa] = S\ cm^{-1} \qquad \text{eq. 3}$$

Ohmic resistances from the conductivity measurement $R_{Electrolyte}$ were corrected by the ohmic resistance of the cables and connections.
Based on the miscibility gap and the measured electrolyte conductivities, an applicable operating range is defined in terms of states of charge (SOC) from 0 % to 100 % in the results chapter "Definition of the operating range for HBr/Br_2 electrolytes".

Solubility of Br_2 in HBr solutions

The solubility curve of bromine in HBr solutions is determined by preparing aqueous HBr solutions of different concentrations c (HBr) between 0 M HBr and 5 M HBr (0, 0.1, 0.25, 0.5, 1, 1.5, 2, 2.5, 3, 3.5, 4, 4.5, 5 M HBr) at room temperature ϑ = 23 °C. Bromine is added dropwise with a pipette. Each drop of Br_2 weighs approximately 25 mg. The total mass of the sample and the change in mass due to the addition of bromine are determined gravimetrically. After adding bromine, the solution is shaken vigorously and left to dissolve completely. If no drop of liquid and heavy bromine remains undissolved in the sample, the solution is not yet saturated with bromine. This procedure is continued iteratively until a drop of bromine remains in the sample. If one drop of bromine remains the sample is saturated with bromine. This can be recognized as a deep brown separate drop which collects at the bottom of the sample due to the high density (ρ) of bromine ρ = 3.1028 g cm^{-3} at ϑ = 25 °C [3]. The addition of bromine increases the

volume and mass of the sample solution. In order to determine the actually existing concentrations at saturation of $c(HBr)_{sat.}$ and $c(Br_2)_{sat.}$, the volume and mass of the samples in the saturated state $V_{sat.}$ and $m_{sat.}$ are measured. The total volume $V_{sat.}$ of the solution is determined volumetrically and the total mass of the sample m_{sat} gravimetrically. By increasing the volume, the concentrations in solution are reduced and can be calculated according to eq. 4 and eq. 5:

$$\mathrm{c(HBr)_{sat.}} = \frac{\mathrm{c(HBr)_0\, V_0}}{\mathrm{V_{sat.}}} \quad \text{eq. 4}$$

$$\mathrm{c(Br_2)_{sat.}} = \frac{\mathrm{m_{sat.} - m_0}}{\mathrm{M_{Br_2}\, V_{sat.}}} \quad \text{eq. 5}$$

V_0 is the volume of the initial HBr solution with the initial concentration $c(HBr)_0$ and its mass m_0. The molar mass of Br_2 is represented by use of M_{Br2}. The solubility curve of the aqueous HBr/Br_2 electrolytes is obtained by plotting the saturation concentration $c(Br_2)_{sat.}$ versus the saturation concentration $c(HBr)_{sat.}$.

Polybromide determination and polybromide distribution by Raman analysis

By means of Raman spectroscopy on the electrolyte samples, the occurrence of the different polybromides Br_3^-, Br_5^-, etc. in aqueous solution in the prepared samples of $HBr/Br_2/H_2O$ is investigated. Raman spectra are measured with the HORIBA LabRAM HR spectrometer (JOBIN YVON Technology GmbH, Germany) at room temperature ϑ = 23 C in the wavenumber ($\tilde{\nu}$) range from $\tilde{\nu}$ = 50 cm^{-1} to 4000 cm^{-1}. A frequency-doubled Nd:YAG laser (neodymium-doped yttrium-aluminium-garnet laser) with an excitation wavelength of λ = 532 nm (green) is used as laser source and a charge coupled device camera detector (CCD) is used at ϑ = -70 °C. Electrolyte samples are pipetted into a quartz glass cuvette (Suprasil QS, Helma), sealed and examined under the microscope. The focal point is positioned just below the edge of the front glass of the cuvette and the position of the carrier table is fixed to obtain reproducible Raman spectra. For all samples and wavenumbers, the exposure time is 10 s and the number of repetitions per sample for calculating the average is set to three.

Simulation of the electrolyte composition

To investigate the validity of polybromide equilibria and equilibrium constants from literature at high reactant concentrations, concentrations of polybromides, HBr and Br_2 in equilibrium are simulated. The corresponding concentrations of Br_2, tribromide and pentabromide ions are calculated for a selected range between 0 M and 7.7 M HBr. To determine global concentrations of Br_2 $c(Br_2)_T$, the stoichiometric relationship, including the global HBr concentration $c(HBr)_T$ and the initial concentration $c(HBr)_0$ = 7.7 M, becomes relevant:

$$c(Br_2)_T = \frac{c(HBr)_0}{2}\left(1 - \frac{c(HBr)_T}{c(HBr)_0}\right) = 3.85\,M\left(1 - \frac{c(HBr)_T}{7.7\,M}\right) \quad \text{eq. 6}$$

The concentrations for $c(Br_3^-)_{eq}$, $c(Br_5^-)_{eq}$, $c(Br_2)_{eq}$, and $c(HBr)_{eq}$ in equilibrium are calculated using eq. 4 and 5 of the main article (K_3 and K_5) with K_3 = 16 L mol^{-1} and K_5 = 37 L^2 mol^{-2} for individual operating SOC points in each case iteratively including eq. 7.

$$\mathrm{c(HBr)_{eq}} = \mathrm{c(HBr)_T} - \mathrm{c(Br_3^-)_{eq}} - \mathrm{c(Br_5^-)_{eq}} \quad \text{eq. 7}$$

The operating points are defined by the state of charge (SOC) of the electrolyte. The SOC range will be defined in chapter “Definition of the operating range for HBr/Br_2 electrolytes”. Concentrations are plotted as a function of SOC in chapters “Theoretical concentrations and polybromide equilibria” and “Polybromide distribution and polybromide concentration verification of polybromide equilibria” both in the main manuscript.

Results of investigation on $HBr/Br_2/H_2O$ electrolytes by helps of Raman spectroscopy

Raman spectra of $HBr/Br_2/H_2O$ electrolytes

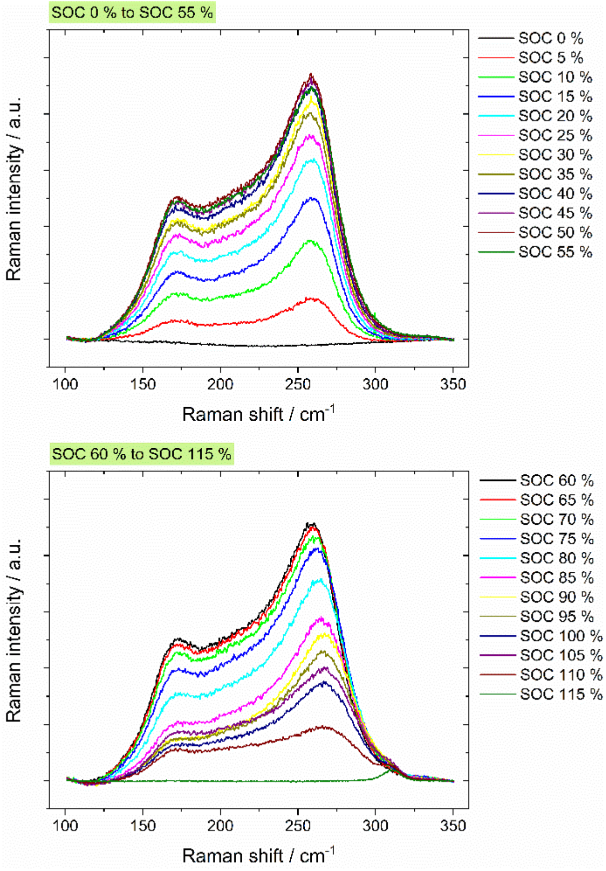

Figure S 1: Raman spectra of different $HBr/Br_2/H_2O$ electrolytes depending on the state of charge SOC (SOC 0 % to SOC 115 %), shown for a Raman shifts between $\tilde{\nu}$ = 100 and 350 cm^{-1}. Selected range for Raman shifts exclusively depicts Raman bands for polybromides and bromine. Mixing conditions of the electrolytes are shown in the manuscript in Table S 1. Raman spectra were corrected for Rayleigh scattering.

Raman spectra of bromine Br_2

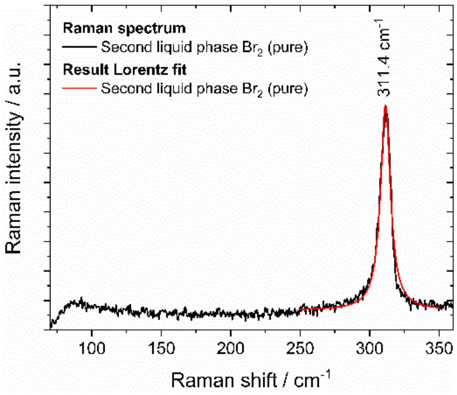

Figure S 2: Raman spectrum of bromine Br_2, shown for a Raman shifts between $\tilde{\nu}$ = 100 and 350 cm^{-1}. Selected range for Raman shifts exclusively depicts Raman bands for polybromides and bromine. Raman spectrum was corrected for Rayleigh scattering. Red line shows the fitting of the spectra by the integration method according to Levenspiel-Marquart with the Lorentz method for symmetrical Raman stretching band of Br_2.

Raman spectra of $HBr/Br_2/H_2O$ electrolytes including fitting of symmetrical and antisymmetrical Raman stretching bands of tri-, penta- and heptabromide

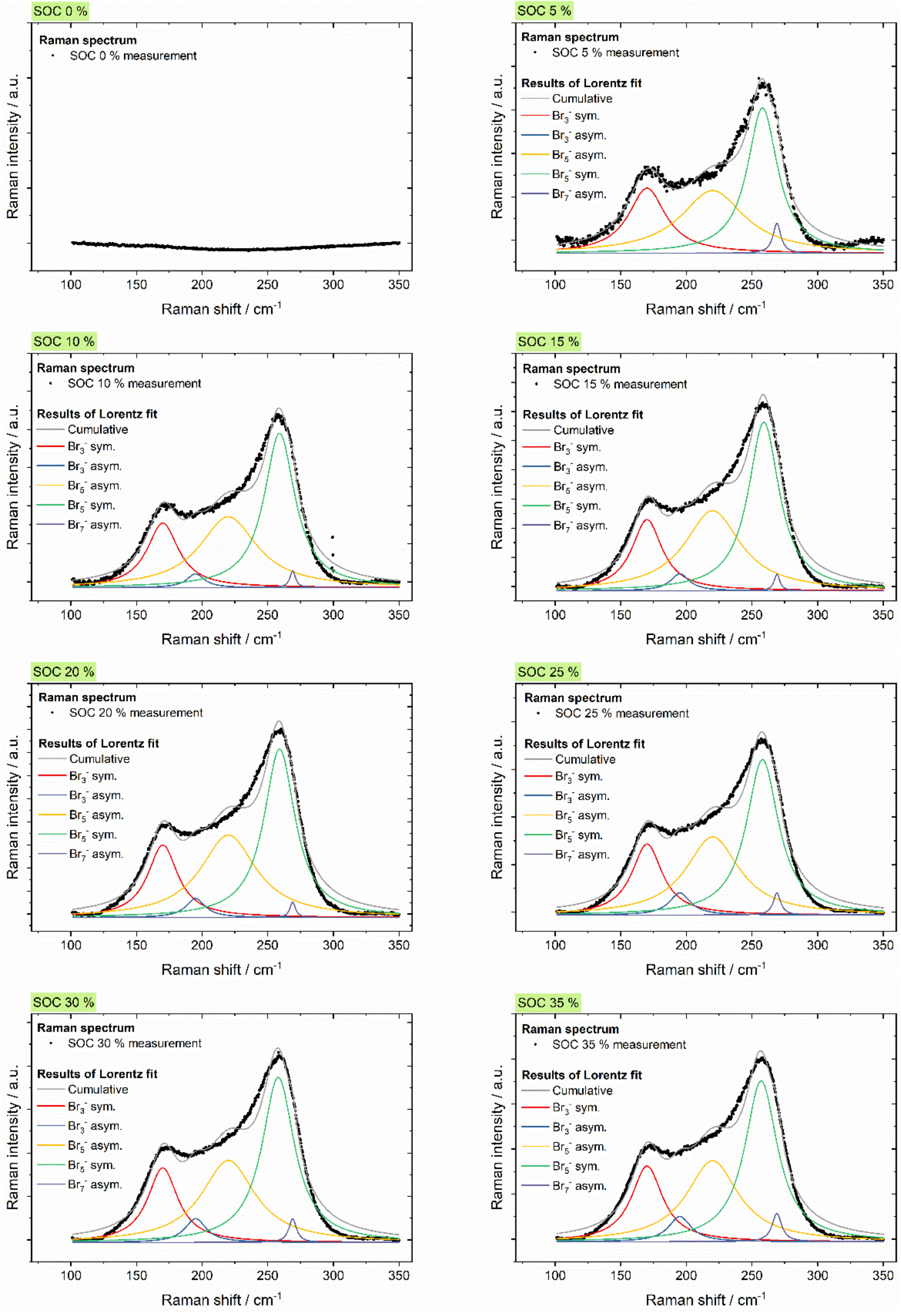

Figure S 3: Raman spectra of different $HBr/Br_2/H_2O$ electrolytes depending on the state of charge SOC (SOC 0 % to SOC 35 %), shown for a Raman shifts between $\tilde{\nu}$ = 100 and 350 cm^{-1}. Selected range for Raman shifts exclusively depicts Raman bands for polybromides and bromine. Mixing conditions of the electrolytes are shown in the manuscript in Table S 1. Raman spectra were corrected for Rayleigh scattering. Coloured lines show the fitting of the spectra by the integration method according to Levenspiel-Marquart with the Lorentz method for symmetrical and asymmetrical Raman stretching bands of the polybromides Br_3^-, Br_5^- and Br_7^-.

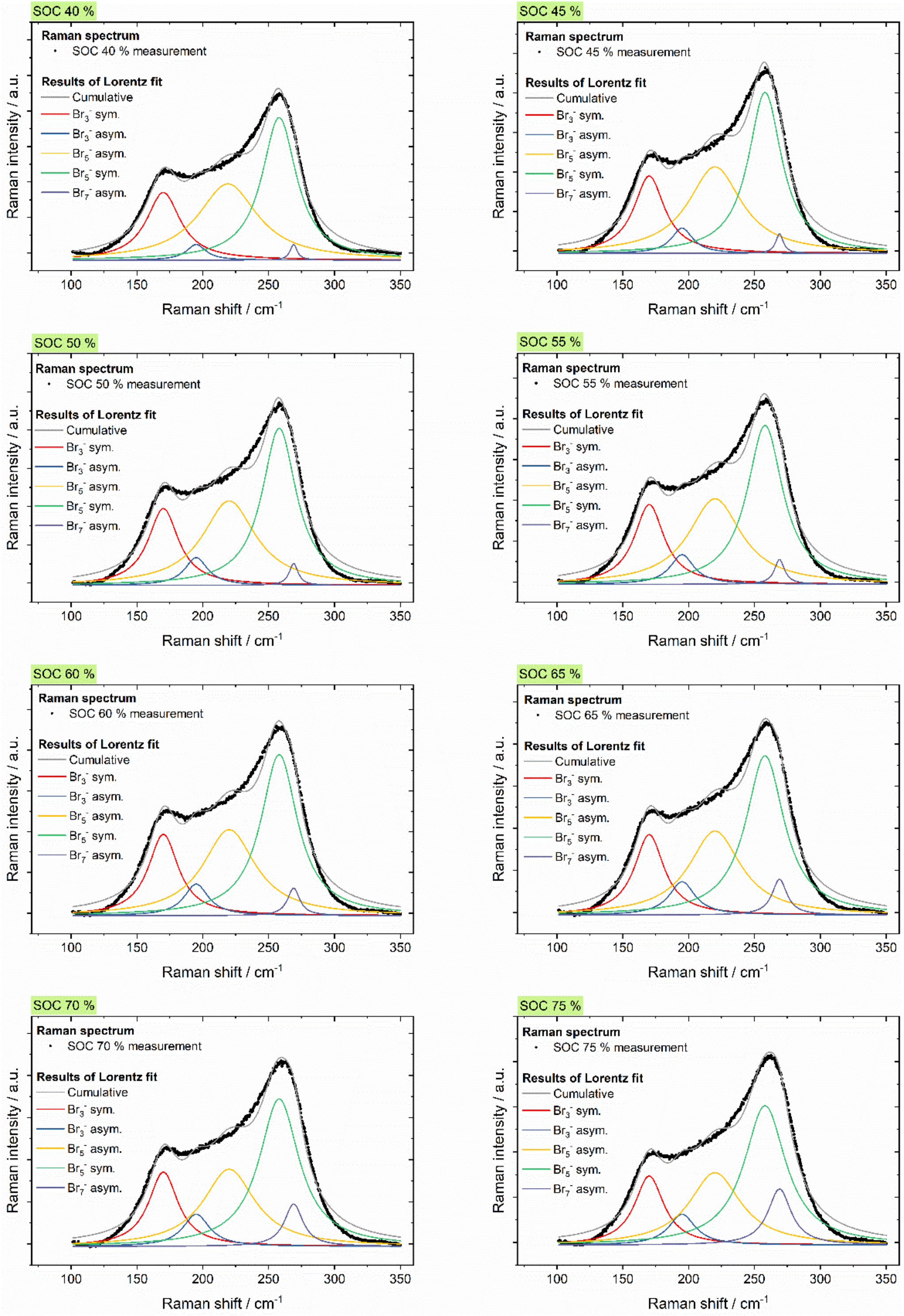

Figure S 4: Raman spectra of different $HBr/Br_2/H_2O$ electrolytes depending on the state of charge SOC (SOC 40 % to SOC 75 %), shown for a Raman shifts between $\tilde{\nu}$ = 100 and 350 cm^{-1}. Selected range for Raman shifts exclusively depicts Raman bands for polybromides and bromine. Mixing conditions of the electrolytes are shown in the manuscript in Table S 1. Raman spectra were corrected for Rayleigh scattering. Coloured lines show the fitting of the spectra by the integration method according to Levenspiel-Marquart with the Lorentz method for symmetrical and asymmetrical Raman stretching bands of the polybromides Br_3^-, Br_5^- and Br_7^-.

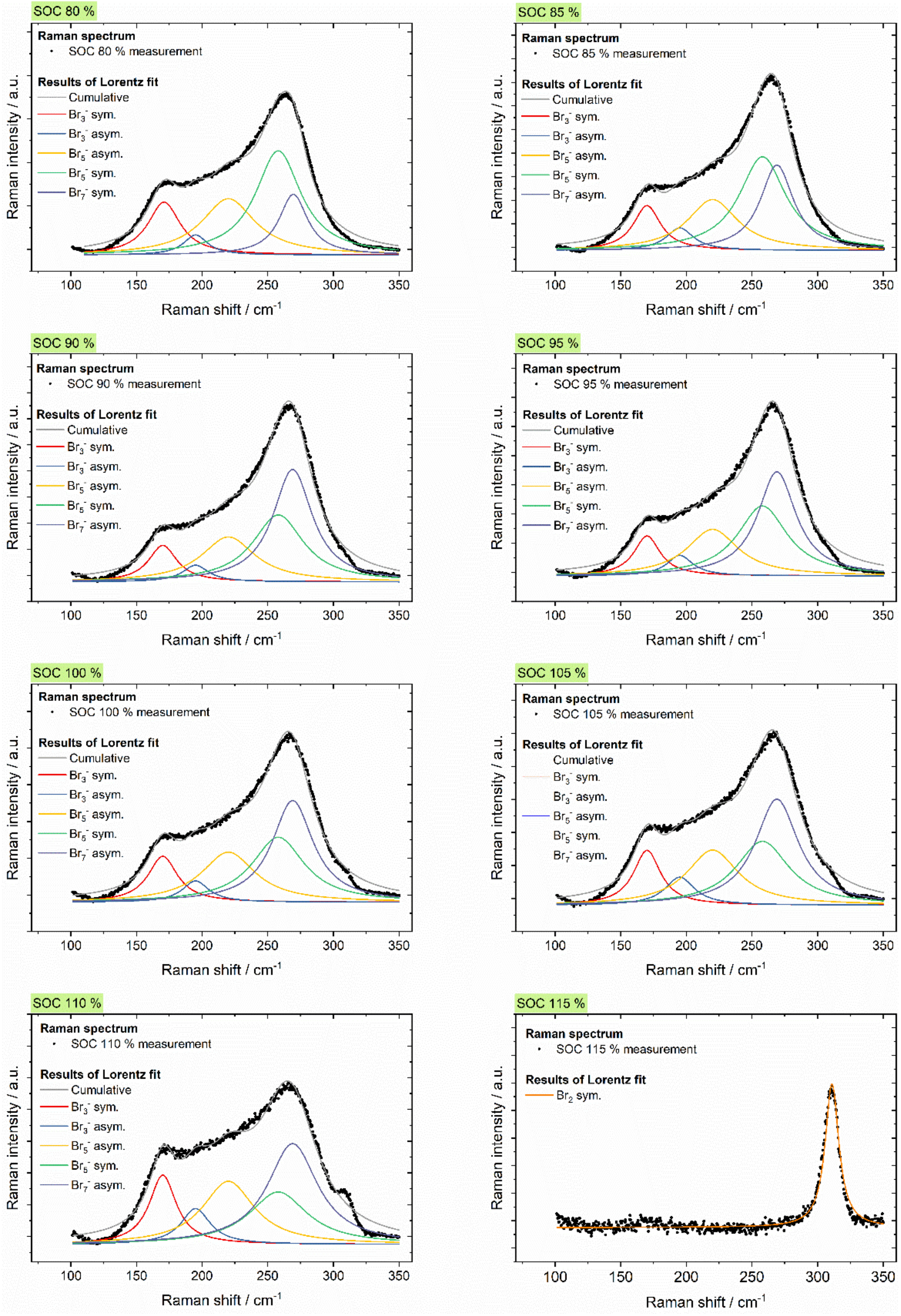

Figure S 5: Raman spectra of different $HBr/Br_2/H_2O$ electrolytes depending on the state of charge SOC (SOC 80 % to SOC 115 %), shown for a Raman shifts between $\tilde{\nu}$ = 100 and 350 cm^{-1}. Selected range for Raman shifts exclusively depicts Raman bands for polybromides and bromine. Mixing conditions of the electrolytes are shown in the manuscript in Table S 1. Raman spectra were corrected for Rayleigh scattering. Coloured lines show the fitting of the spectra by the integration method according to Levenspiel-Marquart with the Lorentz method for symmetrical and asymmetrical Raman stretching bands of the polybromides Br_3^-, Br_5^- and Br_7^-. For SOC ≥ 100 % at $\tilde{\nu}$ = 311 cm^{-1} an overlapping becomes visible which can be assigned to elemental bromine. A second phase of bromine is already present here due to the solubility limit. Bromine is also present in small amounts in the aqueous solution.

Table S 1: Assignment of concentrations of HBr and Br_2 of the electrolyte to the respective SOC and the sample number.

Sample no.	SOC	$c(Br_2)_T$	$c(HBr)_T$
	%	mol L^{-1}	mol L^{-1}
1	0	0	7.700
2	5	0.168	7.365
3	10	0.335	7.030
4	15	0.502	6.695
5	20	0.67	6.360
6	25	0.838	6.025
7	30	1.005	5.690
8	33	1.106	5.489
9	40	1.340	5.020
10	45	1.508	4.685
11	50	1.675	4.350
12	55	1.843	4.015
13	60	2.010	3.680
14	66	2.211	3.278
15	70	2.345	3.010
16	75	2.513	2.675
17	80	2.680	2.340
18	85	2.848	2.005
19	90	3.015	1.670
20	95	3.183	1.335
21	100	3.350	1.000
22	105	3.518	0.665
23	110	3.685	0.330
24	115	3.850	0

Table S 2: Distribution of bromine to the polybromides $x(Br_3^-)$, $x(Br_5^-)$ and $x(Br_7^-)$ and concentrations of the polybromides $c(Br_3^-)$, $c(Br_5^-)$ and $c(Br_7^-)$ in aqueous electrolyte solution.

Sample no.	SOC	$x(Br_3^-)$	$x(Br_5^-)$	$x(Br_7^-)$	$c(Br_3^-)$ / M	$c(Br_5^-)$ / M	$c(Br_7^-)$ / M
1	0	-	-	-	-	-	-
2	5	0.343	0.623	0.034	0.057	0.052	0.002
3	10	0.296	0.691	0.014	0.099	0.116	0.002
4	15	0.289	0.698	0.013	0.145	0.175	0.002
5	20	0.290	0.699	0.011	0.194	0.234	0.003
6	25	0.295	0.683	0.023	0.247	0.286	0.006
7	30	0.290	0.687	0.023	0.291	0.345	0.008
8	33	0.300	0.666	0.034	0.331	0.368	0.013
9	40	0.299	0.683	0.018	0.401	0.457	0.008
10	45	0.301	0.680	0.019	0.453	0.513	0.010
11	50	0.296	0.679	0.025	0.496	0.569	0.014
12	55	0.288	0.681	0.030	0.531	0.628	0.019
13	60	0.283	0.679	0.039	0.568	0.682	0.026
14	66	0.265	0.676	0.059	0.585	0.747	0.044
15	70	0.247	0.664	0.089	0.580	0.778	0.069
16	75	0.221	0.637	0.143	0.554	0.800	0.120
17	80	0.187	0.592	0.220	0.502	0.794	0.197
18	85	0.146	0.518	0.337	0.414	0.737	0.320
19	90	0.117	0.384	0.499	0.340	0.555	0.481
20	95	0.123	0.409	0.468	0.273	0.455	0.347
21	100	0.151	0.381	0.467	0.249	0.314	0.256
22	105	0.157	0.361	0.482	0.173	0.200	0.178
23	110	0.187	0.307	0.506	0.115	0.094	0.103
24	115	-	-	-	-	-	-

References

1 X.-Z. Yuan, Electrochemical impedance spectroscopy in PEM fuel cells. Fundamentals and applications, Springer, London, 2010.

2 W. M. Haynes and D. R. Lide, eds., CRC handbook of chemistry and physics. A ready-reference book of chemical and physical data, CRC Press, Boca Raton, Fla., 96th edn., 2015.

3 D. R. Lide, ed., CRC handbook of chemistry and physics. A ready-reference book of chemical and physical data, CRC Press, Boca Raton, 85th edn., 2004.
